# Engineered g-C_3_N_5_-Based Nanomaterials for Photocatalytic Energy Conversion and Environmental Remediation

**DOI:** 10.3390/nano13030499

**Published:** 2023-01-26

**Authors:** Juanjuan Liu, Shuaijun Wang, Chaocheng Zhao, Jingtang Zheng

**Affiliations:** 1 State Key Laboratory of Petroleum Pollution Control, China University of Petroleum (East China), Qingdao 266580, China; 2 Shandong Engineering and Technology Research Center for Ecological Fragile Belt of Yellow River Delta, Binzhou University, Binzhou 256600, China; 3 School of Energy and Power Engineering, Jiangsu University, Zhenjiang 212013, China

**Keywords:** nitrogen-rich graphitic carbon nitride, defect engineering, heterojunction, energy conversion, environmental remediation

## Abstract

Photocatalysis plays a vital role in sustainable energy conversion and environmental remediation because of its economic, eco-friendly, and effective characteristics. Nitrogen-rich graphitic carbon nitride (g-C_3_N_5_) has received worldwide interest owing to its facile accessibility, metal-free nature, and appealing electronic band structure. This review summarizes the latest progress for g-C_3_N_5_-based photocatalysts in energy and environmental applications. It begins with the synthesis of pristine g-C_3_N_5_ materials with various topologies, followed by several engineering strategies for g-C_3_N_5_, such as elemental doping, defect engineering, and heterojunction creation. In addition, the applications in energy conversion (H_2_ evolution, CO_2_ reduction, and N_2_ fixation) and environmental remediation (NO purification and aqueous pollutant degradation) are discussed. Finally, a summary and some inspiring perspectives on the challenges and possibilities of g-C_3_N_5_-based materials are presented. It is believed that this review will promote the development of emerging g-C_3_N_5_-based photocatalysts for more efficiency in energy conversion and environmental remediation.

## 1. Introduction

As civilization has developed, energy crises and environmental contaminations have become major obstacles to the further development of human society [1,2,3,4]. Semiconductor-based photocatalysis is regarded as one of the most efficient technologies to address serious energy and environmental issues [5,6,7]. The mass production of highly efficient, dependable, and reasonably priced photocatalysts with strong charge carrier segregation, plenty of active sites, a broad optical absorption range, and high redox potentials is crucial for the commercialization of photocatalytic technology [8,9,10]. As a result, much effort has been devoted to designing more robust and efficient photocatalysts.

Graphitic carbon nitride (g-C_3_N_4_) has sparked extensive interest because of its metal-free nature, facile accessibility, excellent physicochemical stability, and appealing electronic band structure [5,6,11]. However, the inherent disadvantage of carrier recombination and the restricted light absorption range dramatically hampered the practical application of g-C_3_N_4_ [12,13]. To elevate the photocatalytic performance of g-C_3_N_4_, versatile modification procedures, such as doping, heterojunctions, and chemical structural regulation (tuning the C/N ratio), have been implemented [14,15]. Compared with g-C_3_N_4_, g-C_3_N_5_ has piqued particular interest because of its nitrogen-rich moiety and sp^2^ hybridized atoms, which assist in the optimization of the electronic band structure [16].

The researchers have recently synthesized 2D g-C_3_N_5_ with different structures using diverse precursors. For example, g-C_3_N_5_ with heptazine moieties linked together by azo linkage (−N=N−) was synthesized by thermal deamination of 2,5,8-trihydrazino-s-heptazine [17]. Terminal triazole-based g-C_3_N_5_ was fabricated via straightforward self-condensation of 3-amino-1,2,4-triazole [18,19,20], while g-C_3_N_5_ with one-triazole and two-triazine combinations was formed via self-assembly of 5-amino-1H-tetrazole (5-ATTZ) [21]. Furthermore, a variety of techniques for modifying g-C_3_N_5_ have been developed, including element doping, defect engineering, and heterojunction engineering. To date, g-C_3_N_5_-based materials have been widely employed in energy conversion and environmental remediation as a new style of carbon nitride specimen [17,22,23,24]. However, a comprehensive review of g-C_3_N_5_-based photocatalysts for energy and environmental applications is still lacking.

This review summarizes the latest progress of g-C_3_N_5_-based photocatalysts for energy and environmental applications. Various synthesis topologies of pristine g-C_3_N_5_ and several engineering strategies for g-C_3_N_5_ (such as elemental doping, defect engineering, and heterojunction creation) are presented. In addition, the photocatalytic applications in H_2_ evolution, CO_2_ reduction, N_2_ fixation, NO purification, and aqueous pollutant degradation are discussed. To the best of our knowledge, this is the first review of g-C_3_N_5_-based photocatalysts. It is anticipated that this review will provide readers with a deep understanding of emerging g-C_3_N_5_-based photocatalysts, which might promote the design of more robust metal-free photocatalysts.

## 2. Synthesis Strategies of Pristine g-C_3_N_5_

**Figure 1:**
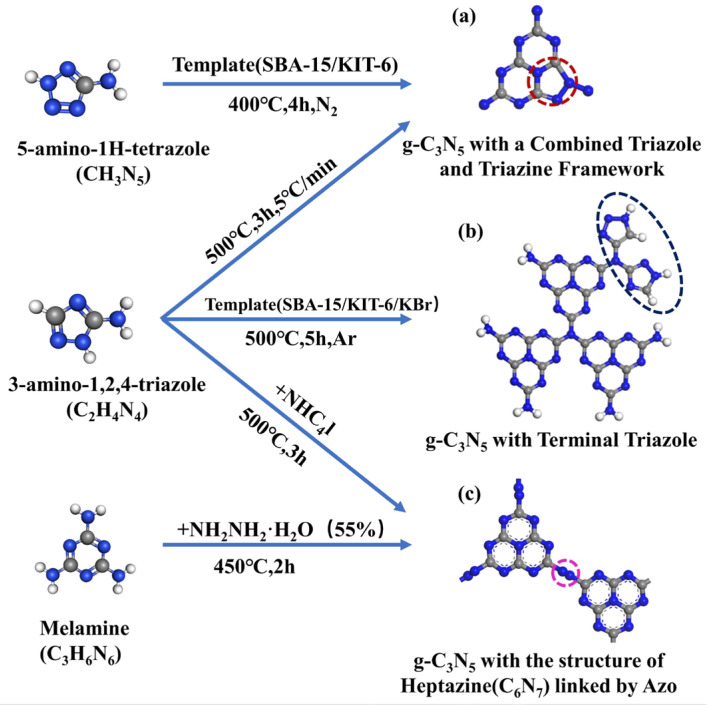
Schematic illustration of the g-C_3_N_5_ synthesis process by the thermal polymerization of different precursors, such as 5-amino-1H-tetrazole, 3-amino-1,2,4-triazole, and melamine. (**a**) g-C_3_N_5_ with triazine and triazole; (**b**) g-C_3_N_5_ with terminal triazole; (**c**) g-C_3_N_5_ with heptazine linked by azo.

In this section, the synthesis techniques and mechanisms of pristine g-C_3_N_5_ with various structures derived from various precursors will be thoroughly explored. The pristine g-C_3_N_5_ can be divided into three types, i.e., g-C_3_N_5_ with triazine and triazole (Figure 1a), g-C_3_N_5_ with terminal triazole (Figure 1b), and g-C_3_N_5_ with azo-linked heptazine (Figure 1c). They can be synthesized by one-step polymerization of inexpensive nitrogen-rich precursors such as 5-amino-1H-tetrazole [21], 3-amino-1,2,4-triazole [18,19,25], and melamine [17].

### 2.1. g-C_3_N_5_ Containing One-Triazole and Two-Triazine

Unlike g-C_3_N_4_, which has three triazine moieties, g-C_3_N_5_ has one triazole and two triazine moieties, with one triazole ring replacing one triazine ring in g-C_3_N_4_. Compared with triazine or heptazine moieties, the triazole moiety possesses more electrons and more pyrrolic N sites than triazine or heptazine, which provides more numbers of the basic sites and enhances the basic catalytic activity. In this section, we will describe the templated and template-free methods for such g-C_3_N_5_ preparations.

#### 2.1.1. The Hard Template Approach

**Figure 2:**
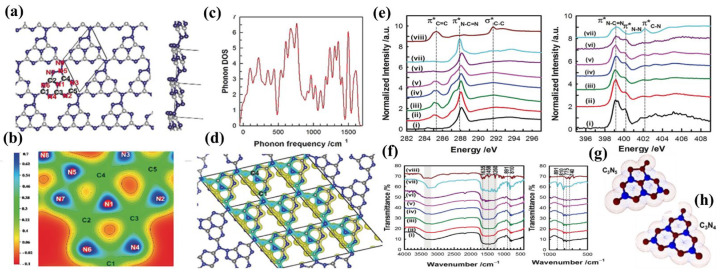
Theoretical and experimental results of g-C_3_N_5_ containing one-triazole and two-triazine: (**a**) Optimized geometric structure (gray for C atoms and blue for N atoms); (**b**) 2D charge density distribution; (**c**) Phonon DOS; (**d**) 3D charge density (yellow regions: electron-rich areas, cyan regions: electron-deficient areas); (**e**) Carbon K-edge (left) and nitrogen K-edge (right) NEXAFS spectra (the black line indicates MCN-11); (**f**) FT-IR spectra (the black line indicates MCN-11); (**g**) g-C_3_N_5_ containing 1-NH_2_/NH-1,2,4-triazole units; (**h**) g-C_3_N_4_ containing 2-NH_2_/NH-1,3,5-triazine units. (**a**–**f**) Adapted with permission from [21]. Copyright Wiley-VCH Verlag GmbH and Co. KGaA, Weinheim, 2018. (**g**–**h**) Adapted with permission from [26]. Copyright WileyVCH GmbH, 2020.

According to the literature, KIT-6 and SBA-15 are two commonly used templates for the synthesis of g-C_3_N_5_. Vinu and co-workers prepared porous g-C_3_N_5_ (MCN-11) via a simple self-assembly of the 5-amino-1H-tetrazole (5-ATTZ) in the presence of hard template KIT-6. In brief, 5-ATTZ was impregnated into the pore channels of the KIT-6 template, carbonized for 4 h at 400 °C, and the template was removed with 5% HF acid to obtain MCN-11. It was demonstrated that the structure of MCN-11 was composed of one triazole and two triazine moieties by DFT calculations (Figure 2a–d) and spectroscopic analyses (Figure 2e,f) [21]. Additionally, the impact of temperature on the material’s nitrogen content, structure, and surface functional groups was also investigated. As the carbonization temperature ascended from 350 to 550 °C, the C/N ratio increased from 1.43 to 1.62, representing the structural change from g-C_3_N_5_ to g-C_3_N_4_. g-C_3_N_5_ containing 1-NH_2_/NH-1,2,4-triazole units (MCN-11) (Figure 2g) was obtained at 350 and 400 °C, whereas g-C_3_N_4_ containing 2-NH_2_/NH-1,3,5-triazine units (Figure 2h) was obtained at 450, 500, and 550 °C [26]. Vinu’s group also synthesized g-C_3_N_5_ nanorods with a combination of triazine and triazole groups, named MCN-14-X, using SBA-15 as a hard template instead of KIT-6. The preparation process is briefly summarized as follows: 5-ATTZ was impregnated into the porosity canals of the SBA-15 template, then carbonized for 4 h at 400 °C, and the template was etched with 5% HF acid to obtain MCN-14 [27]. The obtained mesoporous carbon nitrides, MCN-11 and MCN-14, have a good effect on the adsorption and conversion of CO_2_ and are the catalysts for Knoevenagel condensation. However, this hard template still suffers from cumbersome template preparation processes and an unfriendly template removal process.

#### 2.1.2. The Template-Free Approach

**Figure 3:**
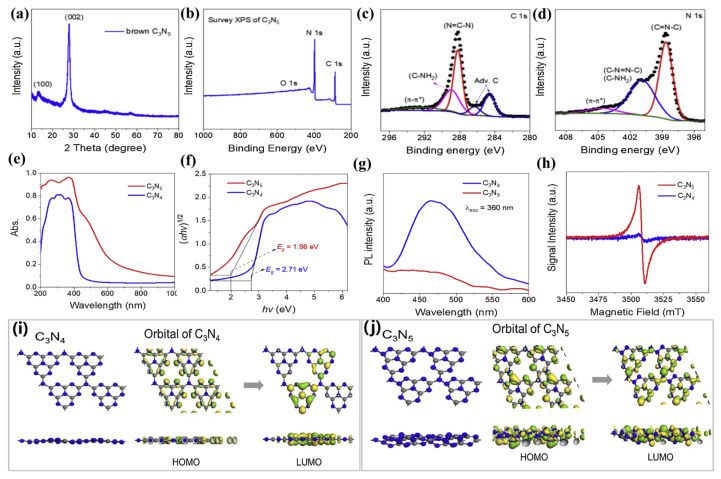
The characterization of g-C_3_N_5_: (**a**) XRD pattern; (**b**) XPS survey; (**c**) high-resolution C1s spectra; (**d**) high-resolution N1s XPS spectra for g-C_3_N_5_; (**e**) UV–vis DRS; (**f**) Tauc’s plot; (**g**) PL spectra; (**h**) ESR spectra for g-C_3_N_5_ and g-C_3_N_4_; (**i**) and (**j**) The orbitals for g-C_3_N_4_ and g-C_3_N_5_. Adapted with permission from [28]. Copyright Elsevier B.V., 2021.

A template-free approach was reported by Zhang et al. [28]. Two-dimension metal-free g-C_3_N_5_ nanosheets were acquired by the polymerization of 3-AT at 500℃ for 3 h under a half-cover air using the heating rate of 5 °C min^−1^. The prepared g-C_3_N_5_ exhibits excellent crystal properties, which can be confirmed by the peaks of 100 (in-plane structural ordering) and 002 (interlayer structural ordering) in XRD characterization (Figure 3a). XPS spectra (Figure 3b–d) confirmed the presence of one-triazole and two-triazine. Compared with g-C_3_N_4_, g-C_3_N_5_ exhibits a better visible light response (Figure 3e), a lower band gap (Figure 3f), and a more effective separation of photogenerated electrons and holes (Figure 3g). It may be due to the abundance of unpaired electrons on g-C_3_N_5_, confirmed by the ESR spectra (Figure 3h). Meanwhile, the orbital calculations (Figure 3i,j) also confirm that g-C_3_N_5_ has better photo-generated carrier separation efficiency than g-C_3_N_4_. Therefore, the prepared g-C_3_N_5_ displays better photocatalytic performance than g-C_3_N_4_. This approach is simple, time-saving, energy-saving, and environment-friendly, which makes it conducive to large-scale production and application of materials.

### 2.2. g-C_3_N_5_ with Terminal Triazole

The structure of g-C_3_N_5_ with terminal triazoles is constructed by 1,2,4-triazoles and 1H-1,2,3-triazoles, which replace two hydrogens on the terminal amino moiety of one triazine ring in g-C_3_N_4_. This replacement both enhanced conjugation and increased nitrogen content (-NH_2_, -NH), which was beneficial to expand the photo-absorption range and enhancing the selectivity and adsorption of acidic gases, respectively. In this section, we will summarize the methods for producing terminal triazole-based g-C_3_N_5_ using KIT-6 and KBr templates.

#### 2.2.1. The Method Using the KIT-6 Template

**Figure 4:**
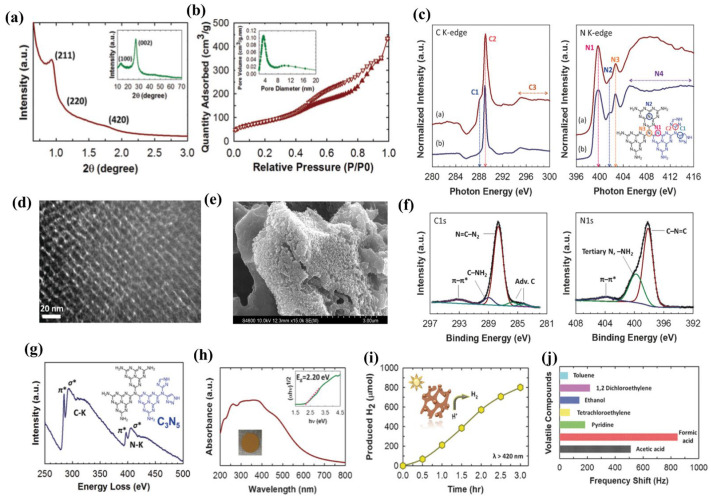
Structural characterization and applications of MCN-8: (**a**) Low-angle powder XRD pattern (inset: wide-angle powder XRD pattern); (**b**) N_2_ adsorption-desorption isotherm; (**c**) Carbon K-edge and nitrogen K–edge NEXAFS spectra (red line for MCN-8, blue line for g-C_3_N_4_, and the inset for the proposed molecular structure); (**d**) TEM image; (**e**) FE-SEM images; (**f**) High-resolution XPS spectra: C1s and N1s; (**g**) EEL spectrum; (**h**) UV–Vis absorption spectrum; (**i**) H_2_ evolution amount in visible light; (**j**) Gas adsorption detected by the QCM sensor. Adapted with permission from [18]. Copyright Wiley-VCH Verlag GmbH and Co. KGaA, Weinheim, 2017.

Vinu et al. [18] have achieved a remarkable breakthrough in the synthesis of mesoporous g-C_3_N_5_, which was named MCN-8. It was synthesized by a thermal condensation process of the 3-amino-1,2,4-triazole precursor in a KIT-6 silica template using a nano-casting technique. In order to detect the structures and properties, advanced characterization techniques are used. XRD measurements reveal the structural ordering and graphitic character of MCN-8 (Figure 4a). According to the low-angle powder XRD pattern, the MCN-8 materials have a highly organized three-dimensional porous mesostructure. The diffraction peak at 2θ = 27.28 (002) verifies the CN framework’s strong crystallinity and interlayer turbostratic ordering, as does a faint peak at 2θ = 13.48 (100), which confirms the in-plane structural ordering. The TEM (Figure 4d) and SEM (Figure 4e) images clearly demonstrate MCN-8’s lattice stripes and porous structure. The N_2_ adsorption-desorption measurement (Figure 4b) also reveals that the MCN-8 has organized mesopores and a large specific surface area, both of which contribute to improved adsorption. The near-edge X-ray absorption fine structure (NEXAFS) (Figure 4c), high-resolution XPS (Figure 4f), and energy loss (EEL) spectra (Figure 4g) demonstrate the existence of the terminal triazole unit and tri-s-triazine ring in the CN framework. This chemical structure not only reduces the bandgap to 2.2 eV (Figure 4h), which is crucial for extending visible light absorption for a faster rate of H_2_ evolution (Figure 4i), but it also demonstrates improved selective sensing of toxic acid molecules (Figure 4j). However, the fabrication of the nano-template (KIT-6) requires a high temperature (540 °C) and a long time, which consumes both energy and time. In order to save energy, Vinu and co-workers [19] used ethanol washing instead of high-temperature calcination to obtain the KIT-6 for synthesizing 3D mesoporous g-C_3_N_5_ (MCN-8E). It was found that the porous structure and graphite essence of MCN-8E are strikingly similar to those of MCN-8. Considering the use of a hazardous etching chemical (HF) for template removal causes the loss of active sites and degrades photocatalytic efficacy as well as the cumbersome preparation of the template KIT-6, the search for an energy-saving, non-toxic, and environment-friendly synthesis route has attracted the attention of researchers.

#### 2.2.2. The Method Using the KBr Template

**Figure 5:**
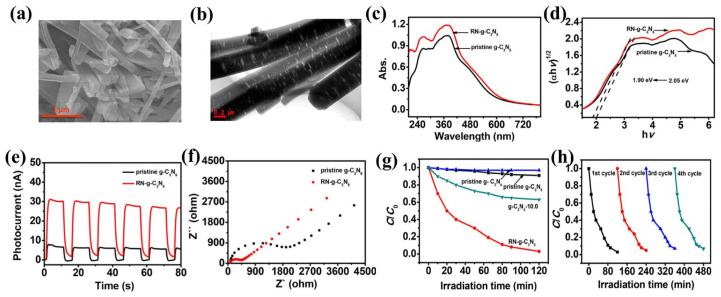
RN-g-C_3_N_5_: (**a**) SEM image; (**b**) TEM image; (**c**) UV–vis DRS spectra; (**d**) Tauc’s plots; (**e**) The photocurrent response; (**f**) EIS spectra; (**g**) MB degradation; (**h**) Recyclability of g-RN-C3N5. Adapted with permission from [20]. Copyright American Chemical Society, 2019.

Greener synthesis approaches are urgently needed to eliminate the use of the toxic HF etchant in g-C_3_N_5_ production. Wang et al. [20] fabricated g-C_3_N_5_ with mesoporous and rod-like morphology (RN-g-C_3_N_5_) via thermal condensation of the 3-amino-1,2,4-triazole with KBr as the template. Figure 5a,b reveal that the prepared RN-g-C_3_N_5_ has a rod-like porous structure due to the guiding effect of KBr. This special structure expands the visible adsorption range (Figure 5c), reduces the bandgap to 1.90 eV (Figure 5d), and improves the separation of electron-hole pairs (Figure 5e,f). As a result, the RN-g-C_3_N_5_ displays superior photocatalytic performance and reusability in MB degradation (Figure 5g,h). It shows greater potential for applications in the field of environmental remediation. Compared with the traditional silica templates, the KBr template is an environmentally friendly strategy for avoiding structural damage, the loss of active sites, and significant environmental contamination caused by HF. In short, this procedure is straightforward, environmentally friendly, and inexpensive.

### 2.3. g-C_3_N_5_ Consisted of Heptazine Units Bridged by Azo

g-C_3_N_5_, consisting of heptazine units (tri−s−triazine) bridged by azo, is an analogue of g-C_3_N_4_, which is derived by replacing the tertiary nitrogen in the g-C_3_N_4_ structure with azo. The extended π−conjugation formed by coupling the orbitals of the azo-linkage with the π-conjugated structures of the heptazine narrowed its bandgap, which expanded the optical absorption range. The theoretical design and experimental fabrication of g-C_3_N_5_ (−N = N−) will be summarized in this section. 

#### 2.3.1. Theoretical Design

Cao et al. [29] designed a 2D crystalline graphitic g-C_3_N_5_ material by employing the heptazine structure as the node and the azo group as the linker. Density functional theory (DFT) was employed to investigate the material’s optical, electrical, and catalytic characteristics. It was found that 2D g-C_3_N_5_ has an excellent performance in non-metal oxygen reduction reactions, gas adsorption, separation, and conversion.

#### 2.3.2. Experimental Fabrication

**Figure 6:**
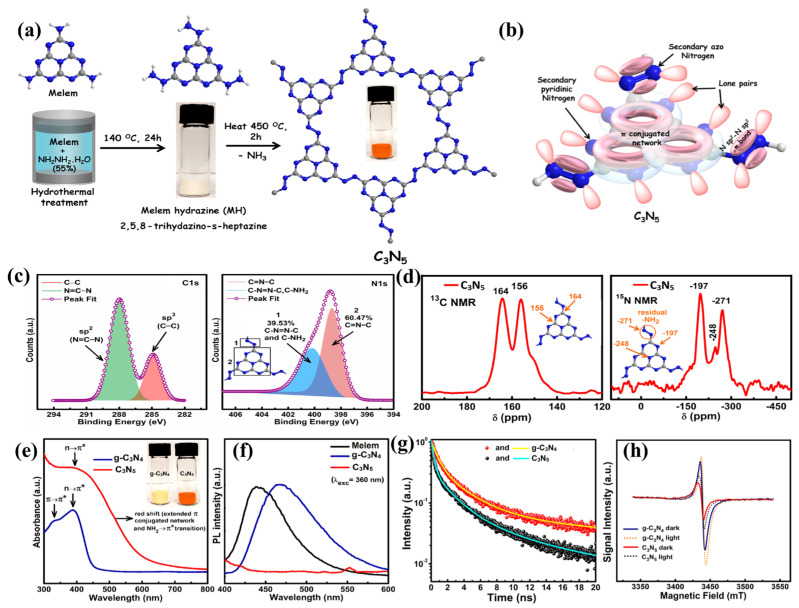
(**a**) Synthesis route; (**b**) molecular orbital overlap representation; (**c**) HR−XPS spectra (left: C1s, right: N1s); (**d**) CPMAS NMR spectra (left: ^13^C, right: ^15^N); (**e**) UV−vis spectra (blue: g−C_3_N_4_; red: g−C_3_N_5_); (**f**) steady state PL spectra; (**g**) PL lifetime decay curves; (**h**) X−band EPR spectra; Adapted with permission from [4]. Copyright American Chemical Society, 2019.

In terms of experimental fabrications, two methods for synthesizing the g-C_3_N_5_ framework are presented. As shown in Figure 6a, Kumar et al. [17] synthesized this g-C_3_N_5_ via thermal deamination of melem hydrazine (MH), also named 2,5,8-trihydrazino-s-heptazine, which was obtained by polymerization of melamine at 425 °C for overnight, followed by a hydrothermal process with hydrazine hydrate. Characterization techniques such as XPS (Figure 6c) and NMR (Figure 6d) indicated that the structure is made up of two s-heptazine units connected by an azo linkage. The overlap of azo atoms with the aromatic network of heptazine units extends conjugation and increases electron density on the heptazine nucleus (Figure 6b). The lower EPR signal strength of g-C_3_N_5_ compared to g-C_3_N_4_ indicates that the number of lone pair electrons in g-C_3_N_5_ drops, confirming that extra N atoms bond with tertiary nitrogen atoms outside the heptazine nucleus to form azo rather than substitute C atoms in the heptazine motif (Figure 6h). This structure increases visible light responsiveness and photogenerated electron-hole separation, as seen by increased visible light absorption in Figure 6e, lower PL intensity in Figure 6f, and a shorter average lifetime of the photogenerated charge carrier in Figure 6g. The 2D g-C_3_N_5_ containing s-heptazine units and azo was successfully prepared by this approach, although the process was complicated and time-consuming. In another work, Liu et al. synthesized g-C_3_N_5_ nanosheets by heating a mixture of NH_4_Cl and 3-amino-1,2,4-triazole (3-AT), followed by protonation exfoliation. It was found that the morphology of the nanosheet increases the specific surface area, facilitates charge separation, and modifies the band structure [30].

In summary, the preparation methods of g-C_3_N_5_ with three different structures, namely, g-C_3_N_5_ with a terminal triazole, g-C_3_N_5_ containing s-heptazine linked by azo, and g-C_3_N_5_ containing 1 triazole and 2 triazines, are presented. It has been witnessed that breakthroughs and progress have been made in the field of g-C_3_N_5_ synthesis, from scratch and complexity to simplicity. It is necessary to further explore more environmentally friendly, energy-saving, time-saving, and labor-saving preparation methods.

## 3. Functional Engineering of g-C_3_N_5_

Pristine g-C_3_N_5_ performs better in the applications of CO_2_ adsorption and conversion, pollutant degradation, and H_2_ evolution. However, pristine g-C_3_N_5_ still suffers electron-hole recombination and limited use of visible light. To address the aforementioned issues, g-C_3_N_5_ functional materials have been designed and fabricated to broaden their uses in a variety of domains. We will go over the preparations of g-C_3_N_5_ functionalized materials in this section, including defect engineering and heterojunction engineering.

### 3.1. Defect Engineering

One strategy for improving the photocatalytic properties of g-C_3_N_5_ is to introduce structural defects into the surface. Defects are produced by the dislocation of an atom, which breaks the periodic arrangement. Doping and vacancy are the two types of engineering defects. Doping usually involves adding atoms to the original structure, while vacancies occur when a host atom is losing its position in the original crystal structure. The defects can cause electronic redistribution in the material, which can regulate spectral absorption and affect the excitation, migration, and recombination of photogenerated electron holes.

#### 3.1.1. Doping Design

Doping is a good strategy because it not only changes the electronic structure, resulting in the band gap and the visible light absorption variation, but it also changes the surface properties, which affect the photocatalytic performance. Next, we will discuss metal doping, non-metal doping, and both metal and non-metal doping in the functional engineering of g-C_3_N_5_.

Metal doping can create defect states in the band structure of g-C_3_N_5_, which trap holes or electrons to change interface charge transfer, inhibiting the recombination of photogenerated holes and electrons. As a new type of N-rich material, the N-rich sites of g-C_3_N_5_ with stronger electronegativity are easy to combine with positive metal ions. Transition metals with unoccupied d orbitals, which may function as electron donors or acceptors, can easily form complexes with g-C_3_N_5_ through electron transfer. The feasibility of transition metal doping g-C_3_N_5_ is verified by Niu’s team [31] through first-principles calculations. They designed a set of models for depositing transition metal (TM) atoms (V, Cr, Mn, Fe, Co, Ni, Cu, Mo, Ru, Pd, W, and Pt) on g-C_3_N_5_ to construct transition metal (TM) doped g-C_3_N_5_ single-atom catalysts (SACs), TM-g-C_3_N_5_. The adsorption energies calculated by DFT are all negative, which demonstrates that the structures of all 12 TM-g-C_3_N_5_ are stable. This study has significant implications for future research into g-C_3_N_5_-based stable photocatalytic materials.

**Figure 7:**
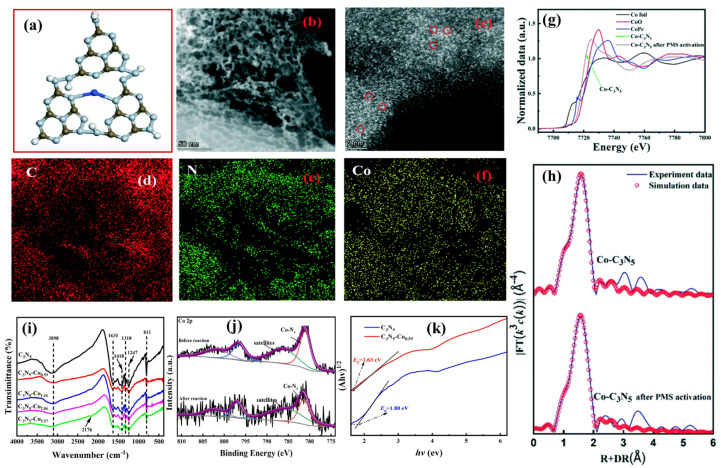
Co-C_3_N_5_ (**a**) Doping structure; (**b**) HR-TEM image; (**c**) HAADF-STEM images; (**d**–**f**) elemental mapping; (**g**) XANES spectra; (**h**) R-space of XANES; (**i**) FT-IR spectra; (**j**) XPS spectra of Co_2p_; (**k**) Tauc’s plot. (**a**–**h**) Adapted with permission from [32]. Copyright Royal Society of Chemistry, 2022. (**i**–**k**) Adapted with permission from [33]. Copyright Elsevier B.V., 2022.

In terms of material fabrication, Fang et al. [32] prepared pristine g-C_3_N_5_ according to Zhang’s method [28] and pyrolyzed a mixture of it with potassium chloride, lithium chloride, and cobalt chloride dihydrate to prepare cobalt-doped g-C_3_N_5_, whose doping pattern is shown in Figure 7a. HR-TEM images (Figure 7b), HAADF-STEM images (Figure 7c), and elemental mapping (Figure 7d–f) all show that individual nitrogen atoms are uniformly doped into g-C_3_N_5_. In addition, it was also confirmed that the configuration of Co-N_4_ was formed by the combination of Co with pyridine nitrogen in g-C_3_N_5_, as confirmed by the XANES spectra (Figure 7g) and R-space of XANES (Figure 7h). The prepared Co-C_3_N_5_ has stable catalytic performance for PCB28 degradation, which is attributed to sulfate radicals from PMS activated by Co-N_4_. Similar work has also been done by Gan et al. [33]. They prepared pristine g-C_3_N_5_ according to Vinu’s method [18] and pyrolyzed the mixture of pristine g-C_3_N_5_ with 1,10-phenanthroline monohydrate and (CH₃COO)₂Co·4H₂O to prepare another cobalt-doped g-C_3_N_5_. The structure of Co-N binding was confirmed by advanced characterization methods, such as a peak at 2170 cm^−1^ in FT-IR spectra (Figure 7i) and a Co-Nx peak in the XPS spectra of Co_2p_ (Figure 7j). Cobalt doping significantly enhances optical properties, such as reducing the band gap from 1.80 to 1.63 eV in Tauc’s plot (Figure 7k), which helps to activate PMS for the generation of active species, including free radicals (·O_2_^−^, SO^−^_4_·, and ·OH) and non-free radicals (^1^O_2_ and tetravalent cobalt oxide). In SMX degradation experiments, it can be degraded with a removal rate of 99.57% in 20 min. The tetravalent cobalt oxide performed a vital role, whereas the other species made a minor contribution. In addition, Vinu’s team [34] synthesized mesoporous titanium carbonitride, named MTiCN. It was obtained by carbonizing the mixture of mesoporous C_3_N_5_ and titanium tetrachloride at 900 °C for 5 h. The resulting MTiCN samples have a rod-like morphology, a mesoporous structure, and a large specific area, as well as a high carbon content. It shows excellent H_2_ evolution performance similar to commercial platinum/carbon. Moreover, Vivek Polshettiwar [35] synthesized K-doped g-C_3_N_5_ (K-g-C_3_N_5_) via the thermal polymerization of 3-AT and KBr. A dipole interaction is generated between K and N atoms as opposed to K atoms replacing N atoms, hence altering the distribution of electrons and narrowing the band gap to increase light absorption. As an electron trap, K also facilitates the transfer and separation of photogenerated carriers.

Non-metallic doping is another effective method of modifying g-C_3_N_5_. Since it has a high electron affinity and electronegativity, it may easily substitute the N atom in g-C_3_N_5_ and establish covalent bonds with the C atom. Non-metallic ions penetrate the lattice and act as impurity energy levels in the valence band, therefore reducing the band gap, separating electrons and holes, and enhancing the catalytic properties. To date, non-metallic components such as N, B, P, etc. have been employed to modify g-C_3_N_5_.

**Figure 8:**
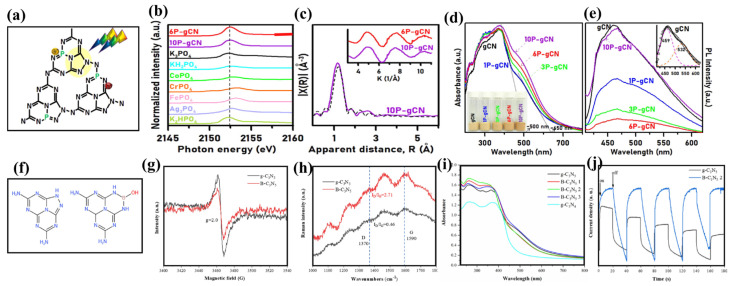
(**a**) Doping structure of P-C_3_N_5_; (**b**) K-edge XAFS spectra of 6P-gCN, and 10P-gCN samples and phosphorus reference compounds; (**c**) FT of the k^2^-weighted EXAFS spectrum (purple line) and the curve fitting result for the 10P-gCN sample (dotted line). Inset shows the k^2^-weighted EXAFS spectra; (**d**) UV-vis; (**e**) PL spectra; (**f**) A schematic illustration of B-C_3_N_5_; (**g**) ESR spectra; (**h**) Raman spectra; (**i**) UV-vis DRS spectra; and (**j**) the transient photocurrent response. (**a**–**e**) Adapted with permission from [36]. Copyright American Chemical Society, 2021. (**f**–**j**) Adapted with permission from [37]. Copyright Elsevier B.V., 2022.

Hu et al. [36] investigated the phosphorus doping of g-C_3_N_5_ (P-g-C_3_N_5_). P-doped triazole-based g-C_3_N_5_ was produced by thermal condensation at 500 °C for 4 h, followed by thermal copolymerization at 500 °C for another 4 h, using 3-AT and HCCP as precursors. Figure 8a depicts its doping structures: P−N/P = N structures were formed between P and N, which were derived from the XAFS spectra (Figure 8b) and EXAFS spectra (Figure 8c). P_2P_ orbitals provide donor levels in the forbidden band to donate more electrons, narrowing the band gap and increasing the visible light usage rate, as confirmed by UV-vis spectra (Figure 8d). Compared with the undoped g-C_3_N_5_ specimen, P-g-C_3_N_5_ demonstrated a higher separation efficiency of photogenerated charges, which was confirmed by PL spectra (Figure 8e). As a result, the P-g-C_3_N_5_ displays an excellent rhodamine B and tetracycline degradation rate.

Li et al. [38] constructed 18 varieties of B-doped g-C_3_N_5_, including substitution doping and pore doping, followed by investigating the impact of B-doping on the material’s photo-absorption range, band structure, and charge separation. The results revealed that the optimal impurity levels increased light absorption, minimized the recombination of photogenerated charge, and improved the performance of the catalyst. In terms of material preparation, Qu et al. [37] synthesized boron-doped g-C_3_N_5_ (B-C_3_N_5_) by a one-step pyrolysis process employing ammonium borate and 3-AT, whose structure is shown in Figure 8f. The increased I_D_/I_G_ (Figure 8h) shows that the graphite-like structure was distorted, confirming the B as the heteroatom doped into the structure of the CN. Indeed, the decreased C/N ratio calculated by element analysis confirmed the loss of carbon atoms. The doping of B makes up for the loss of the C atom, as confirmed by the decreased ESR spectra intensity of B-C_3_N_5_ (Figure 8g). That is to say, B tends to occupy the C vacancy sites in C_3_N_5_ to form the B-O-H functional group, which can expand the light absorption range (Figure 8i), enhance the charge transfer ability (Figure 8j), and optimize the structural stability. In the photocatalytic nitrogen fixation experiments, it can achieve a photocatalytic nitrogen fixation rate of 421.18 μmol·h^−1^·g^−1^, demonstrating its superior performance. The B-O-H groups play the most important role; B can absorb and activate nitrogen using its Lewis acid characteristics, and O-H can provide H protonate to activated nitrogen for NH_3_ formation using its Bronsted acidic characteristics.

Ding et al. [39] synthesized an N-doped carbon catalyst (PDA-g-CN) by pyrolyzing g-C_3_N_5_@PDA hybrid, which was created by the polymerization reaction of dopamine hydrochloride and g-C_3_N_5_ under mild alkaline conditions, at 800 °C for 2 h. The developed PDA-g-CN catalyst has shown good degradation efficiency for organic pollutants (SMX) via the non-radical mechanism. Nitrogen with a higher electronegativity makes the surface potential of carbon more positive, thereby enhancing the adsorption capacity of PMS and promoting the formation of high-redox potential C-PMS* complexes, which can oxidize organic contaminants (SMX) via electron transfer from SMX to C-PMS*.

Metal and nonmetal doping play a significant role in the modification of g-C_3_N_5_, which can alter the band gap, light absorption, and photogenerated charge separation. Most metal elements may enter the lattice by pore doping, whereas most non-metal elements may enter via substitution doping. On top of that, Ao et al. [40] synthesized the metal K and nonmetal I co-doped g-C_3_N_5_ (K, I-g-C_3_N_5_) via pyrolyzing 3-AT and KI. K may enter the lattice by pore doping, and I may enter via substitution doping. The band gap of K and I co-doped g-C_3_N_5_ is larger than that of pristine g-C_3_N_5_, which is unfavorable for light absorption. Fortunately, the more negative conduction band potential has a strong reduction ability and can reduce O_2_ to produce H_2_O_2_ (O_2_ + 2e^–^ + 2H^+^ → H_2_O_2_). It was also confirmed that the co-doping of metal and non-metal is an effective way to improve the properties of g-C_3_N_5_.

#### 3.1.2. Vacancy Engineering

**Figure 9:**
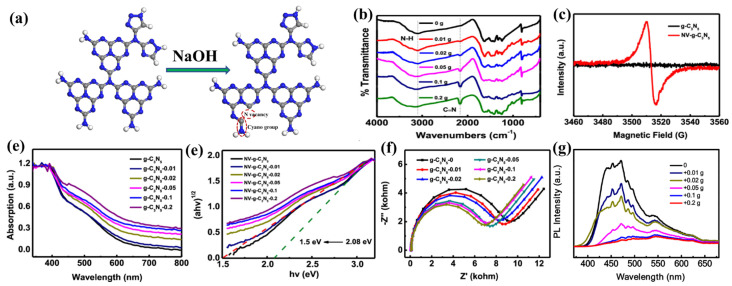
(**a**) Structures of g-C_3_N_5_ and N_V_-g-C_3_N_5_; (**b**) FT-IR spectra; (**c**) ESR spectra; (**d**) UV-vis spectra; (**e**) Tauc’s Plots; (**f**) EIS spectra; (**g**) FL spectra of g-C_3_N_5_ and N_V_-g-C_3_N_5_ (0.01, 0.02, 0.05, 0.1, and 0.2 represent the amounts of NaOH for vacancy production). (**b**–**f**) Adapted with permission from [41]. Copyright American Chemical Society, 2020.

Vacancy defects also play an important role in enhancing photocatalytic capacity, which can be summarized as having three important aspects: light absorption ability, carrier separation efficiency, and surface reaction ability. In the field of materials, nitrogen vacancies and oxygen vacancies are commonly used to regulate photocatalytic performance. For example, Wang et al. [25] fabricated a triazole-based g-C_3_N_5_ material with N vacancies (Nv-g-C_3_N_5_) through the thermal polymerization of 3-AT assisted by NaOH. Later, Zhang et al. [41] used this method to fabricate the same Nv-g-C_3_N_5_ for constructing the N_V_-g-C_3_N_5_/BiOBr heterojunction. The structure is shown in Figure 9a. A strong Lorentz signal peak observed in the ESR curve of Nv-g-C_3_N_5_ confirmed the existence of nitrogen vacancies (Figure 9c). The peak at 2170 cm^−1^ observed in the FT-IR spectrum suggests the formation of cyano groups (Figure 9b). The cyano-groups extend the photo-absorption range (Figure 9d), reduce the band gaps from 2.08 to 1.5 eV (Figure 9e), and use N vacancies as electron traps to capture and store excited electrons, which facilitate the separation of photogenerated charge carriers. The Nyquist radius of N_V_-g-C_3_N_5_ decreases with the increase of N vacancies in Figure 9f, which is conducive to electron and hole migration and separation. In addition, the PL intensity of Nv-g-C_3_N_5_ decreases with the increase of N vacancies in Figure 9g, also reflecting the enhanced separation rate of charge carriers. Both nitrogen vacancies and cyano groups affect the photocatalytic and photoelectrochemical properties of materials. We believe that this simple, effective, and low-cost vacancy engineering is beneficial to the wide application of g-C_3_N_5_ in energy conversion and environmental remediation.

### 3.2. g-C_3_N_5_-Based Heterojunctions

**Figure 10:**
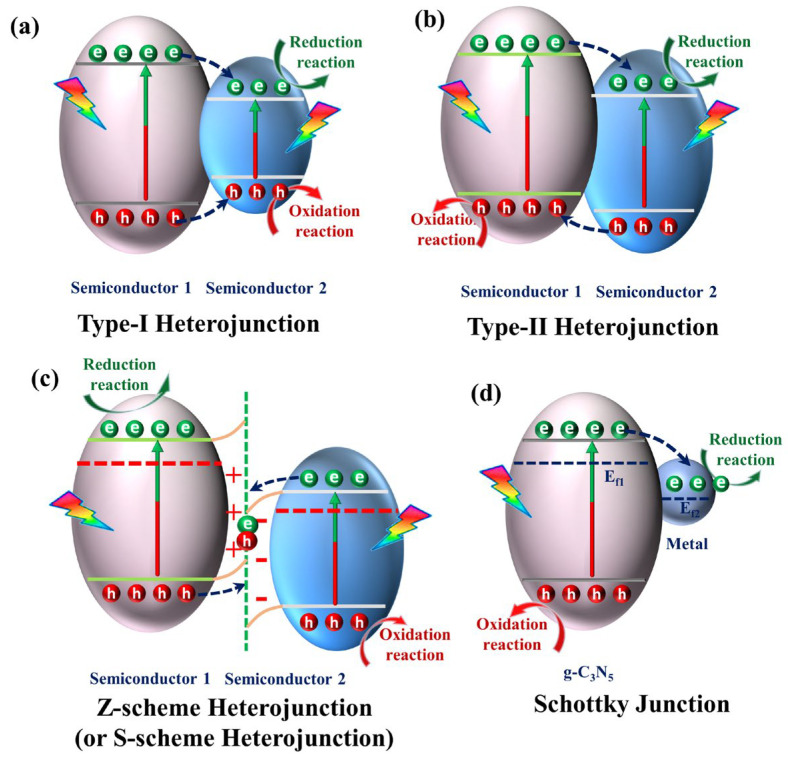
Schematic illustration of photoinduced carrier transfer on different heterojunctions: (**a**) type-I heterostructure; (**b**) type-II heterostructure; (**c**) Z-scheme heterostructure or S-scheme heterostructure; (**d**) Schottky junction.

Constructing heterojunctions between the two semiconducting materials is an efficient strategy for facilitating charge transfer. The photocatalytic activity of g-C_3_N_5_ materials can be boosted by constructing heterojunctions with another semiconductor. Until now, many g-C_3_N_5_-based heterostructures have been synthesized for energy conversions and environmental governance, such as g-C_3_N_5_/BiOBr [41], Bi_4_O_5_Br_2_/g-C_3_N_5_ [42], Bi_4_O_5_I_2_/g-C_3_N_5_ [43], Bi_2_WO_6_/g-C_3_N_5_ [44], Bi_2_MoO_6_/g-C_3_N_5_ [45], CeTi_2_O_6_/g-C_3_N_5_ [46], Ag_3_PO_4_/g-C_3_N_5_ [47], Ag_2_CO_3_/g-C_3_N_5_ [48], AgCl/g-C_3_N_5_ [49], FeOCl/g-C_3_N_5_ [50], LaCoO_3_/g-C_3_N_5_ [51], and MOFs/g-C_3_N_5_ [52]. This section will give a brief overview of heterojunctions based on g-C_3_N_5_, including type-I heterojunction, type-II heterojunction, Z-scheme heterojunction or S-scheme heterojunction, and Schottky junction. Their photoinduced carrier transfer routes are shown in Figure 10.

#### 3.2.1. Type-I Heterojunction

**Figure 11:**
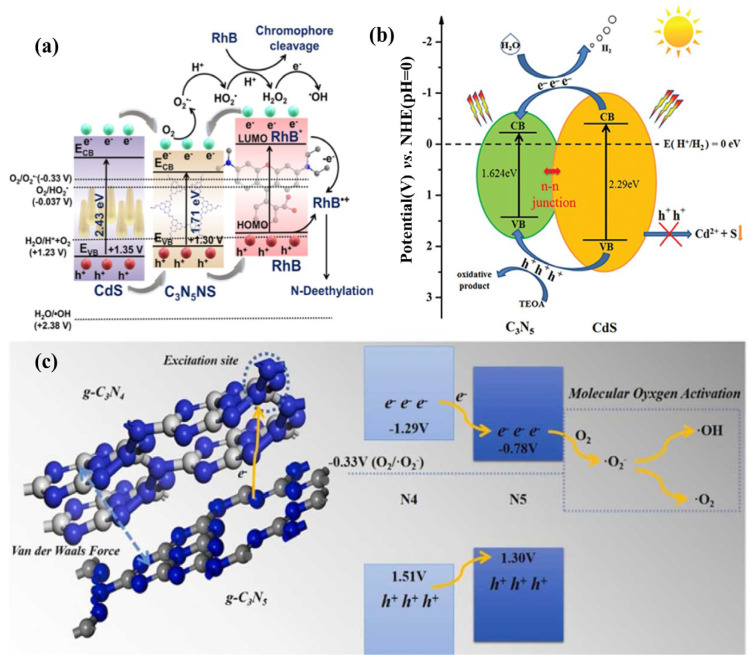
(**a**) CdS/C_3_N_5_ for RhB degradation; (**b**) CdS/C_3_N_5_ for H_2_ evolution; (**c**) g-C_3_N_4_/g-C_3_N_5_ for RhB and TC-HCl. (**a**) Adapted with permission from [53]. Copyright American Chemical Society, 2021. (**b**) Adapted with permission from [54]. Copyright Informa UK Limited, trading as Taylor and Francis Group, 2022. (**c**) Adapted with permission from [55]. Copyright Elsevier B.V., 2022.

For a type-I heterojunction (Figure 10a), the energy band structure is nested, with the bottom of the conduction band and the top of the valence band of the narrow band material situated inside the forbidden band of the wide band material. Both holes and electrons are transmitted from semiconductor 1 to semiconductor 2, where they may combine. Consequently, this heterojunction restricts the separation of electrons and holes. There are few materials about type-I heterojunction among the g-C_3_N_5_-based heterojunctions. It is worth noting that the azo and heptazine rings expand the conjugation of g-C_3_N_5_, increasing the migration properties and separation efficiency of electrons and holes. Thus, g-C_3_N_5_ can be used to improve another semiconductor property via type-I heterojunction. For example, Alam et al. [53] fabricated CdS/C_3_N_5_ heterojunctions with a core-shell structure by wrapping CdS nanowires with g-C_3_N_5_ shells obtained via melamine thermal deamination. It greatly outperformed CdS nanorods alone in the removal of 4-nitrophenol and rhodamine B (RhB) (Figure 11a), which was attributed to the passivating effect generated by the g-C_3_N_5_ shell for CdS and the effective charge separation produced by the type-I heterojunctions between CdS and g-C_3_N_5_. Through type-I heterojunction, the electrons transfer from CdS to g-C_3_N_5_, and the holes also transfer from CdS to g-C_3_N_5_, thus promoting hole extraction on CdS. Furthermore, photoinduced electron migration and separation are facilitated by the expanded conjugation in g-C_3_N_5_. In addition, Wang et al. [54] fabricated another kind of CdS/C_3_N_5_ for enhanced H_2_ production. It is fabricated by the hydrothermal reaction of the uniform suspension solution obtained by adding g-C_3_N_5_ to an aqueous solution of NH_2_CSNH_2_ and Cd(NO_3_)_2_·4H_2_O at 180 °C for 12 h. The synthesized CdS/C_3_N_5_ greatly decreased the electron-hole recombination rate via type-I heterojunction, which can produce hydrogen more than four times faster than pristine g-C_3_N_5_ (Figure 11b). Furthermore, Wang et al. [55] successfully fabricated the g-C_3_N_4_/g-C_3_N_5_ VDWs junction via a facile calcination technique with the precursors 3-AT for g-C_3_N_4_ and melamine for g-C_3_N_5_. It exhibited outstanding RhB and TC-HCl degradation performance, which was attributable to the charge transfer from g-C_3_N_4_ nanolayers to g-C_3_N_5_ nanorods through a type-I heterojunction interface and then effectively activating molecular oxygen to generate ROS (Figure 11c).

#### 3.2.2. Type-II Heterojunction

Type-II heterojunction (Figure 10b) has a staggered arrangement of energy bands; the CB and VB potentials of semiconductor 1 are higher than those of semiconductor 2. Under visible light irradiation, photoinduced electrons may migrate from the CB of semiconductor 1 to that of semiconductor 2, while photoinduced holes are transferred in the opposite direction, from semiconductor 2 to semiconductor 1. Photoinduced electrons and holes accumulated on semiconductors 2 and 1, respectively. Consequently, type-II heterojunctions seem to achieve charge carrier separations.

**Figure 12:**
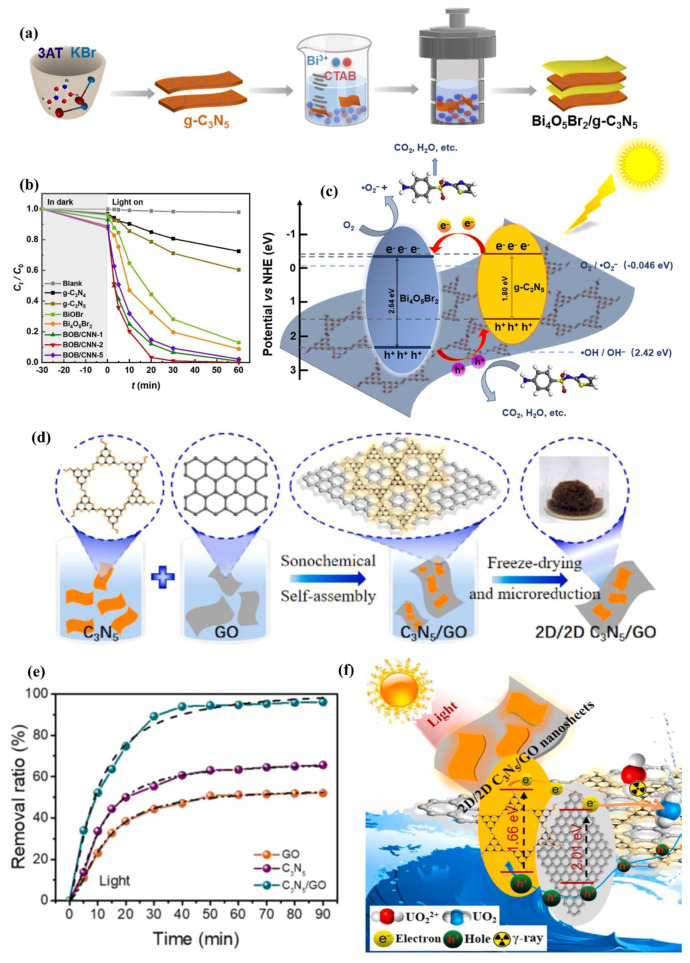
(**a**) g-C_3_N_5_/Bi_4_O_5_Br_2_ preparation routes; (**b**) Photocatalytic degradation of STZ by g-C_3_N_5_/Bi_4_O_5_Br_2_; (**c**) Photoreaction mechanism of STZ by g-C_3_N_5_/Bi_4_O_5_Br_2_; (**d**) Diagram of the g-C_3_N_5_/GO preparation; (**e**) U(VI) removal ratio; (**f**) Mechanism schematic of U (VI) extraction by C_3_N_5_/GO. (**a**–**c**) Adapted with permission from [42]. Copyright Elsevier B.V., 2021 (**d**–**f**) Adapted with permission from [56]. Copyright Elsevier B.V., 2021.

In this section, g-C_3_N_5_-based type-II heterojunction materials and their synthesis methods are briefly reviewed. The basic process is to prepare g-C_3_N_5_ material, then mix it with other materials through precipitation or hydrothermal reaction to obtain the corresponding heterojunction materials. For example, Sun et al. [42] constructed a g-C_3_N_5_/Bi_4_O_5_Br_2_ type-II heterojunction by in-situ growth of Bi_4_O_5_Br_2_ on g-C_3_N_5_ nanosheets through the hydrothermal method (Figure 12a). The interlayer morphology and N-rich structure enhanced visible light utilization, the bismuth-rich property of Bi_4_O_5_Br_2_ increased the excited charge carrier lifetime, and the type-II surface heterojunction (g-C_3_N_5_/Bi_4_O_5_Br_2_) promoted charge transfer and separation. As shown in Figure 12c, the reactive species ·O_2_^−^, produced by the reaction between separated electrons on the CB of Bi_4_O_5_Br_2_ and the adsorbed O_2_, together with the separated holes on the VB of g-C_3_N_5_, improve the degradation efficiency of sulfathiazole (Figure 12b). Indeed, Meng et al. [56] constructed a 2D/2D C_3_N_5_/GO type-II heterojunction using a sonochemical self-assembly method, achieving a U(VI) removal rate of 96.1% (Figure 12d,e). The photoreduction ability of the electrons on the GO conduction band and the physical adsorption ability of GO itself play an important role (Figure 12f). In addition, p-n heterojunction, a special type-II heterojunction, is composed of p-type and n-type semiconductors. Under visible light irradiation, the photoexcited electrons in the conduction band of the p-type semiconductor move to the n-type semiconductor, driven by the built-in electric field, while the holes move in the opposite direction, forming a p-n heterojunction. For example, Zhang et al. [41] used a hydrothermal process to make N_V_-g-C_3_N_5_/BiOBr. Nitrogen vacancies and P-N heterojunction promote N_2_ adsorption and activation.

However, the theory of type-II heterojunctions has also been questioned. Yu et al. [57,58] have presented the inadequacies of the theory of type-II heterojunction from thermodynamic, kinetic, and energy viewpoints. First, the carrier migration to lower CB or higher VB reduces the redox ability of the material accompanying the energy loss. Secondly, this transfer is also kinetically unfavorable because of the repulsive forces between electrons and electrons and holes and holes.

Z-scheme or S-scheme heterojunction (Figure 10c) has the same interleaved energy band structure as a Type-II heterojunction but a completely different mechanism for charge transfer. Electrons migrate from a high Fermi energy level to a low Fermi energy level when two semiconductors come into contact, creating an internal electric field (IEF) and energy band edge bending. Additionally, there are also Coulomb interactions between electrons and holes. As a result of the three aforementioned actions, electrons and holes with low redox potential merge, leaving electrons and holes with high redox potential in the conduction band of semiconducting 1 and the valence band of semiconductor 2. As a result, Z- or S-scheme heterojunctions can achieve excellent separation of photogenerated carriers while retaining a high redox capacity.

Z-scheme heterojunction is developed on the basis of liquid-phase and solid-state Z-scheme heterojunction. Yu et al. [57,58] demonstrated that the charge transfer hypothesis of liquid-phase and all-solid Z-scheme heterostructures is unfavorable in thermodynamics and dynamics, proposing that the “Z-scheme” name be abandoned and substituted with a new name: S-scheme heterostructure. In accordance with the literature, we will discuss the S- and Z-scheme heterostructures, respectively.

#### 3.2.3. Z-Scheme Photocatalyst

**Figure 13:**
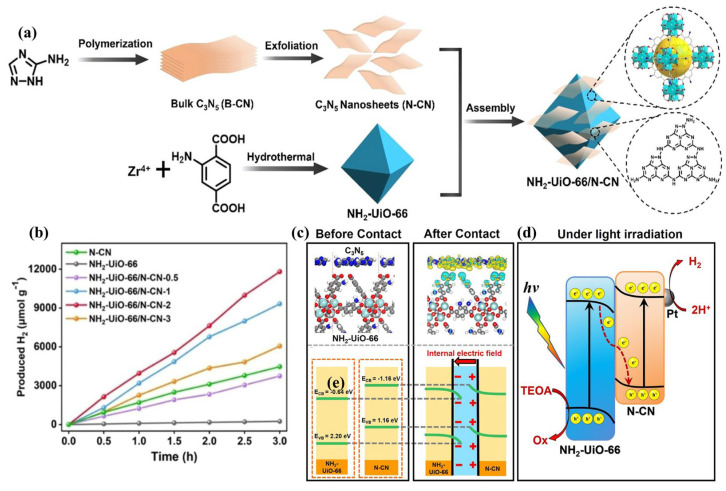
NH_2_-UiO-66/N-CN Z-scheme Heterojunction: (**a**) synthetic routes; (b) photocatalytic H_2_ production amounts; (**c**) charge density differences and energy-level diagrams; (**d**) the proposed Z-scheme charge transfer mechanism. Adapted with permission from [52]. Copyright American Chemical Society, 2022.

Z-scheme heterojunction (Figure 10c) has been shown to be a promising technique for improving a single semiconductor’s photocatalytic efficiency. It can also be employed to modify g-C_3_N_5_ materials without doubt. To enhance the photocatalytic performance of g-C_3_N_5_, C_3_N_5_-based Z-scheme heterojunctions composed of g-C_3_N_5_ and the other semiconductor have been investigated. Herein, g-C_3_N_5_-based Z-scheme heterojunction materials and their synthesis methods are briefly reviewed. The basic process is to prepare the g-C_3_N_5_ material first, then mix it with other materials through liquid phase mixing, in-situ precipitation, hydrothermal reaction, and calcination to obtain the corresponding heterojunction materials. For example, Shi et al. [52] fabricated NH_2_-UiO-66/N-CN Z-scheme heterojunction photocatalysts via assembly in liquid mixing (Figure 13a). Taking Pt as a cocatalyst, the fabricated NH_2_-UiO-66/N-CN-2 displays a high hydrogen evolution rate (Figure 13b), which is attributed to the separation of photo-generated carriers facilitated by the close contact interface of Z-scheme heterojunction. This charge transfer mechanism was also verified by the density functional theory calculation (Figure 13c). This provides an effective method to improve the performance of the g-C_3_N_5_ photocatalyst by adjusting the electron distribution to promote charge separation through heterojunction interface engineering.

#### 3.2.4. S-Scheme Heterojunction

**Figure 14:**
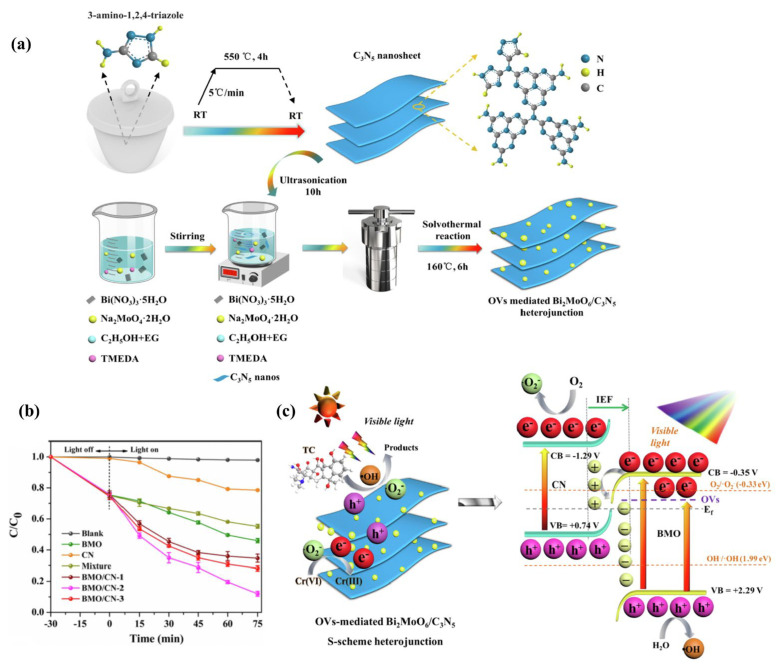
g-C_3_N_5_/Bi_2_MoO_6_ S-scheme heterojunction: (**a**) synthetic routes; (**b**) photocatalytic degradation of TC; (**c**) S-scheme charge transfer mechanism. Adapted with permission from [45]. Copyright Elsevier Inc, 2022.

The reported C_3_N_5_-based S-scheme heterostructure consisting of g-C_3_N_5_ and another oxidizing semiconductor may improve the photocatalytic activity of g-C_3_N_5_. Through charge transfer at the interface of the S-scheme heterojunction, the holes in g-C_3_N_5_ may be coupled with the electrons in another semiconductor through charge transfer at the interface of an S-scheme heterojunction. The electrons in g-C_3_N_5_ and holes in another semiconductor were preserved, which successfully separates electrons and holes and maintains a high redox capability. Herein, g-C_3_N_5_-based S-scheme heterojunction materials and their synthesis methods are briefly reviewed. The basic process is to prepare the g-C_3_N_5_ material first, then mix it with other materials through wet chemical synthesis, hydrothermal reactions, and a solvothermal approach to obtain the corresponding heterojunction materials. For example, Li et al. [45] constructed a Bi_2_MoO_6_/C_3_N_5_ S-scheme heterojunction with oxygen vacancies (OVs) via in-situ solvothermal synthesis (Figure 14a). Benefiting from the efficient separation and transfer of photogenerated charge carriers by S-scheme charge transfer, enriched structural defects, and the close contact interface formed by the in-situ growth, this material remarkably enhanced visible-light photocatalytic degradation efficiencies for TC and Cr(VI) (Figure 14b), whose degradation mechanisms are shown in Figure 14c. The development of S-scheme heterojunctions holds great promise for the design of g-C_3_N_5_-based photocatalysts and the applications of g-C_3_N_5_-based materials in energy conversion and environmental remediation.

#### 3.2.5. Schottky Junction

A Schottky junction is made from a metal and an n-type semiconductor material, similar to a p-n junction. N-type semiconductors have a higher Fermi energy level than metal, so electrons flow from the semiconductor to the metal. This makes an electron accumulation layer on the metal side and an electron depletion layer on the semiconductor side. This creates an electric field from the semiconductor to the metal, or a “Schottky barrier”, permitting electrons to go from the semiconductor to the metal but not return. The metal plays the role of converging electrons and promotes the effective separation of electrons and holes. In this section, we will talk about three types of Schottky materials for g-C_3_N_5_: conventional structures, core-shell structures, and two-dimensional nanosheet structures.

In conventional Schottky connections, metal particles are dispersed or deposited on the semiconductor surface. For example, Zhang et al. [59] fabricated Pt-C_3_N_5_ and P-Pt-C_3_N_5_ by loading Pt on g-C_3_N_5_ through NABH_4_ reduction and in-situ photo-deposition, respectively. Chen et al. [60] constructed a Ni-C_3_N_5_ material by loading Ni onto g-C_3_N_5_ using a simple annealing method. The Schottky barrier allows electrons to pass from the g-C_3_N_5_ conductor band to Pt (Ni) but not back, thereby preventing the combination of photogenerated electrons and holes in g-C_3_N_5_ and enhancing the photocatalytic performance of Pt- and Ni-C_3_N_5_.

**Figure 15:**
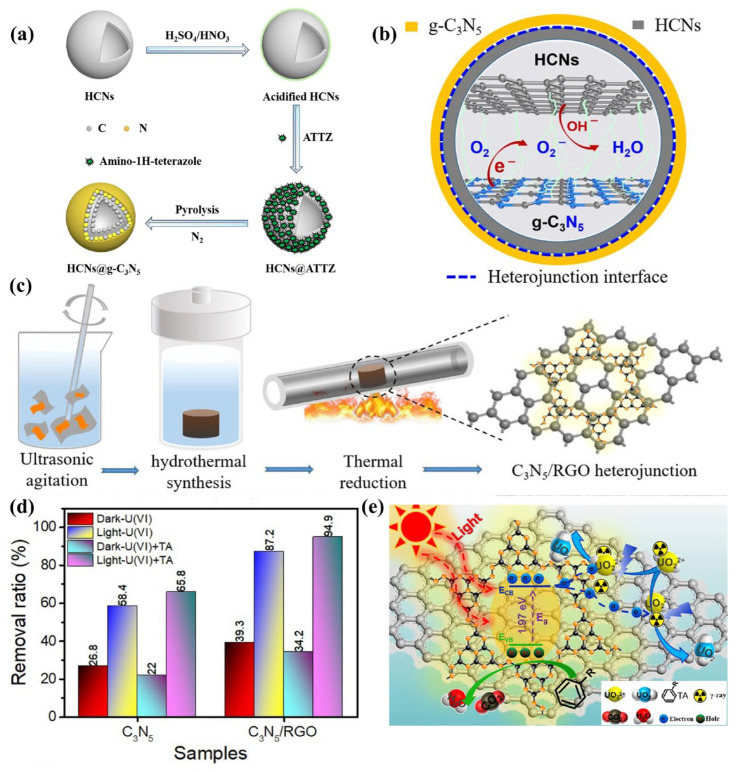
(**a**) Schematic illustration of HCNs@g-C_3_N_5_ synthesis; (**b**) The core-shell structure and proposed charge transfer pathway in HCNs@g-C_3_N_5_; (**c**) Schematic illustration of C_3_N_5_/RGO preparation; (**d**) The U(VI) removal ratio by C_3_N_5_/RGO under different conditions; (**e**) Mechanism schematic of U(VI) extraction by 3D C_3_N_5_/RGO. (**a**−**b**)Adapted with permission from [61]. Copyright Elsevier B.V., 2021. (**c**−**e**) Adapted with permission from [62]. Copyright Elsevier B.V., 2021.

The core-shell structure is formed by wrapping metal nanoparticles with semiconductor materials, thus forming strong contact between two materials at the interface. Li et al. [61] synthesized HCNs@ATTZ by impregnating 5-amino-1H-tetrazole (5-ATTZ) into hollow carbon nanospheres (HCNs), which were subsequently carbonized at 400 °C to produce HCNs@g-C_3_N_5_ (Figure 15a). This is a core-shell-structured Schottky heterojunction in which HCNs are the core and g-C_3_N_5_ is the shell (Figure 15b). The difference in Fermi levels produced band bending and an internal electric field. They act similarly to a Schottky heterojunction to promote the separation of electrons and holes.

Two-dimensional (2D) nanosheet materials with chemically active surfaces or edges have a larger specific surface area, which may provide more active sites for photoelectrochemical reactions compared to pristine particles. Meng et al. [62] fabricated the Schottky heterojunction C_3_N_5_/RGO containing 2D-structured g-C_3_N_5_ and 2D-structured reduced graphene oxide (Figure 15c). Electrons were transported from g-C_3_N_5_ to RGO through a Schottky heterojunction, efficiently separating electrons and holes. Moreover, RGO with high electrical conductivity was favorable for electron migration. As a result, the produced C_3_N_5_/RGO exhibits good photoreduction for uranium (Figure 15d,e).

## 4. Environmental and Energy Applications

g-C_3_N_5_ has been extensively applied in two major areas: energy conversion (H_2_ evolution and N_2_ reduction) and environmental remediation (pollutant degradation, CO_2_ reduction, NO removal, and peroxide activation). They will be discussed in the subsequent chapters.

### 4.1. H_2_ Evolution Reaction (HER)

Solar-driven water-splitting technology has become a research hotspot for sustainable hydrogen manufacturing technology. According to the thermodynamic properties, the process of overall H_2_O spitting for hydrogen and oxygen evolution is non-spontaneous with a Gibbs free energy of 237.2 kJ·mol^−1^, which is equal to a potential of 1.23 eV. For the hydrogen evolution reaction (HER), the potential at the semiconductor’s conduction band bottom must be more negative than the hydrogen evolution potential (H^+^/H_2_ = 0 eV). For the oxygen evolution reaction (OER), the potential at the valence band top must be more positive than the oxygen evolution potential (H_2_O/O_2_ = 1.23 eV). If you want to achieve H_2_ and O_2_ evolutions at the same time, the band gap of the semiconductor photocatalyst must be greater than 1.23 eV. For example, Liu et al. [30] synthesized pristine C_3_N_5_ nanosheets with a band gap of 1.5 eV, a CB potential of −0.333 eV, and a VB potential of 1.217 eV. The conduction band bottom is more negative than 0 eV, which satisfies the hydrogen evolution conditions. The hydrogen evolution rate of 28.97 μmol·g^−1^·h^−1^ was achieved. Although the band gap is greater than 1.23 eV, the valence band potential is less than 1.23 eV (the OER potential), which means OER cannot take place.

In addition, the g-C_3_N_5_ functional materials have also been employed for H_2_ evolution and exhibit excellent performance. For example, NixSy-C_3_N_5_ [63], S-Ni(OH)_2_-C_3_N_5_ [64], Pt-C_3_N_5_ [59], CD/MoS_2_/C_3_N_5_ [65], CdS/C_3_N_5_ [54], CdS/C_3_N_5_(CCN) [66], g-C_3_N_5_/Zn_0_._5_Cd_0_._5_S [67], NH_2_-UiO-66/N-CN-2 [52]. The applications of g-C_3_N_5_-based photocatalyst in H_2_ production are summarized in Table 1.

### 4.2. CO_2_ Reduction Reaction (CO_2_RR)

CO_2_, being the primary byproduct of burning fossil fuels, may considerably contribute to global warming and environmental harm. Photocatalytic CO_2_ reduction reactions (CO_2_RR) are a promising technique that converts CO_2_ to hydrocarbon fuels. It not only lowers CO_2_ contaminants but also supplies clean fuels such as methane (CH_4_), methanol (CH_3_OH), and ethanol (CH_3_CH_2_OH) that can replace fossil fuels. However, because the linear CO_2_ molecule has a relatively stable physical chemistry property with a Gib free energy of 394.28 KJ·mol^−1^, it is difficult to convert CO_2_ into C1 compounds (CO, CH_4_, and CH_3_OH) and C2 compounds (CH_3_CH_2_OH). To break the initial carbon-oxygen bond (750 KJ·mol^−1^), a tremendous amount of energy is required. Due to the remarkable improvement in visible light utilization, g-C_3_N_5_-based materials are expected to be candidates for CO_2_RR. The photogenerated holes can oxidize H_2_O into O_2_ and H^+^, which H^+^ can couple with electrons to reduce CO_2_ to C1 products, followed by coupling C and C to obtain the C2 product. In order to accomplish the above reduction process, the potential at VB must be more positive than that of O_2_/H_2_O, and the potential at CB bottom must be more negative than that of C1/CO_2_.

**Figure 16:**
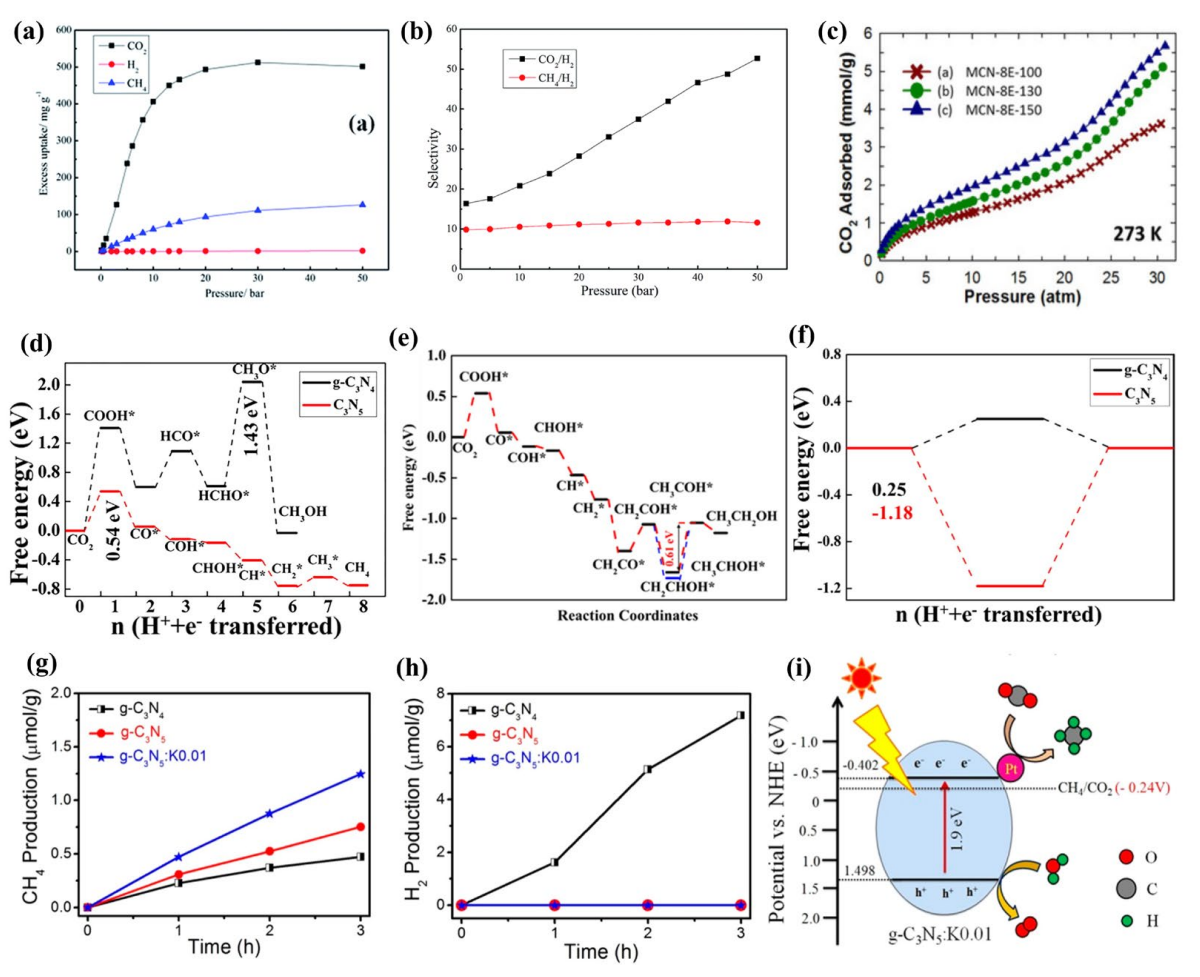
Adsorption and conversions of CO_2_: (**a**) Adsorption performance; (**b**) Adsorption selectivity for the binary mixtures CO_2_/H_2_ (20/80) and CH_4_/H_2_ (50/50); (**c**) CO_2_ adsorption on MCN-8E-T; (**d**,**e**) Calculated free energy diagram corresponding to the optimal path of the CO_2_ conversion to CH_4_ and CH_3_CH_2_OH on the g-C_3_N_5_, respectively; (**f**) Free energy diagram of HER (side reaction) on the g-C_3_N_5_; (**g**) and (**h**) CH_4_ and H_2_ evolution rate in photocatalytic CO_2_ reduction by g-C_3_N_5_: K0.01, respectively; (**i**) Schematic representation of the photocatalytic CO_2_ reduction using g-C_3_N_5_: K0.01. (**a**−**b**) Adapted with permission from [29]. Copyright Royal Society of Chemistry, 2018. (**c**) Adapted with permission from [19]. Copyright Wiley-VCH Verlag GmbH and Co. KGaA, Weinheim, 2017. (**d**−**f**) Adapted with permission from [69]. Copyright Elsevier Inc, 2020. (**g**−**i**) Adapted with permission from [35]. Copyright American Chemical Society, 2022.

**Figure 17:**
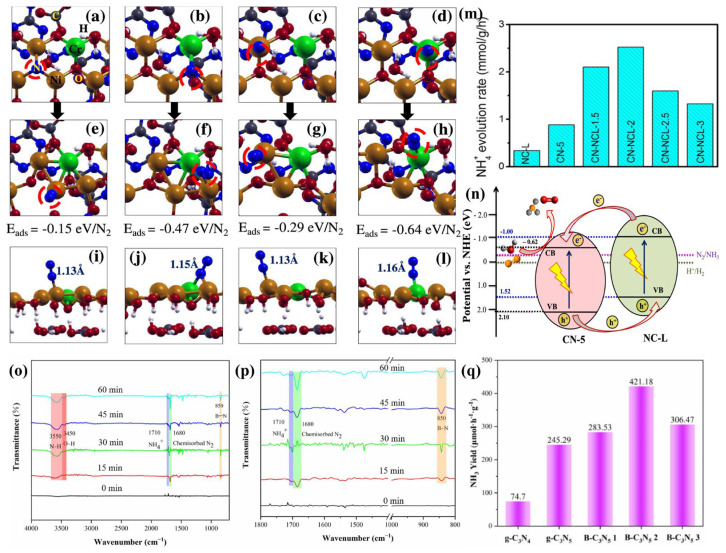
(**a**–**d**) Initial structure (red circles: the binding position); (**e**–**h**) corresponding optimized structures; (**i**–**l**) corresponding N−N bond distances; (**m**) Photocatalytic ammonia production rates of g-C_3_N_5_/NiCr-LDH; (**n**) Schematic illustration of a mechanism for NH_3_ production using g-C_3_N_5_/NiCr-LDH; (**o**–**p**) In-situ DRIFTS spectra of B-C_3_N_5_; (**q**) NH_3_ yields for g-C_3_N_5_ and B-C_3_N_5_ x (x = 1, 2, 3). (**a**–**n**) Adapted with permission from [70]. Copyright Elsevier B.V., 2022. (**o**–**q**) Adapted with permission from [37]. Copyright American Chemical, 2020.

Adsorption is the key step for CO_2_ conversion. Cao et al. [29] demonstrated that g-C_3_N_5_ (−N = N−) with basicity groups exhibits preferable affinity and notable adsorption selectivity for CO_2_ by DFT calculation. At 298 K, the adsorption capacities of CO_2_, CH_4_, and H_2_ are 11.8 mmol g^−1^ at 30 bar, 7.9 mmol g^−1^ at 50 bar, and 0.75 mmol g^−1^ at 50 bar, respectively (Figure 16a). It indicated that g-C_3_N_5_ is a viable choice for the adsorptive separation of CO_2_/H_2_ and CH_4_/H_2_, particularly CO_2_/H_2_ (Figure 16b). Vinu et al. [19] prepared the ordered mesoporous g-C_3_N_5_ (MCN-8E-T) for CO_2_ adsorption with 5.63 mmol g^−1^ of capacity at 273 K and 30 bar (Figure 16c). Vinu’s colleagues [27] also investigated MCN-14 materials with different pore sizes, which exhibit 5.6–9.1 mmol g^−1^ of CO_2_ adsorption abilities at 0 °C and 30 bar and 14–38% of Faraday efficiencies for CO formation. Except for this conversion from CO_2_ to CO, Morikawa et al. [69] demonstrated that g-C_3_N_5_ (-N = N-) can efficiently catalyze CO_2_ to CH_4_ and CH_3_CH_2_OH under visible light illumination by DFT calculation, with −0.54 eV (Figure 16d) and −0.61 eV (Figure 16e) of limiting potentials, respectively. Both of them were less than HER free energy (1.18 eV) (Figure 16f), which indicated that g-C_3_N_5_ is an effective catalyst for CO_2_RR due to its strong suppressing effect on HER. Bharati Debnath and coworkers [35] reported that 1% K-doped g-C_3_N_5_ (-N = N-) could convert CO_2_ to CH_4_ with H^+^ provided by H_2_O, displaying 100% selectivity (Figure 17g–i). The keys to improving CO_2_ absorption and reduction may be a large specific surface area, the suitable size of pores, a more negative conduction potential with strong reduction capacity, and effective charge separation.

### 4.3. Nitrogen Reduction Reaction (NRR)

Inspired by the process of biological nitrogen fixation, photocatalytic nitrogen reduction for ammonia synthesis has garnered attention. As the g-C_3_N_5_ is excited by photons, the generated holes can oxidize H_2_O into O_2_ and H^+^, and electrons may activate N_2_ with the aid of H^+^ to generate NH_3_. Compared with the Haber-Bosch method, this progressive hydrogenation method avoids energy waste and CO_2_ emissions, which is both energy-saving and environmentally friendly. The potential at the CB bottom needs to be more negative compared to the N_2_/reduction products (N_2_/N_2_·, N_2_/N_2_H, N_2_/N_2_H_2_, N_2_/N_2_H_4_, N_2_/N_2_H_5_^+^, and N_2_/NH_3_), while the potential at VB top needs to be more positive compared to that of O_2_/H_2_O.

Bharati Debnath [70] designed a g-C_3_N_5_/NiCr-LDH heterostructure, a hybrid of g-C_3_N_5_ and NiCr-layered double hydroxide. DFT calculations showed its ability to adsorb and activate N_2_ molecules. The possible adsorption configurations and binding sites, the optimized configurations and adsorption energy (Eads), and the N–N bond lengths of the adsorbed N_2_ molecules are presented in Figure 17a–d, Figure 17e–h, and Figure 17i–l, respectively. The lesser absorption energy (−0.64 eV/N_2_) confirmed that the Cr atom is the best adsorption site of the N_2_ molecule. The increased N–N bond length (1.16 Å) compared to the free N_2_ molecule (1.078Å) suggests the activation of the N_2_ molecule. The experimental results also confirmed that the outstanding NH_3_ yields of g-C_3_N_5_/NiCr-LDH were 7.51 and 2.86-folds larger than those of pristine g-C_3_N_5_ and NiCr-LDH, respectively (Figure 17m). This is mainly due to the separation of electrons and holes produced by the heterojunction interface between g-C_3_N_5_ and NiCr-LDH, which facilitates more electrons for reducing N_2_ to NH_3_ (Figure 17n).

B-C_3_N_5_, produced by doping boron into g-C_3_N_5_, was used to immobilize N_2_ for NH_3_ synthesis, as confirmed by in-situ DRIFTS spectra (Figure 17o–p), with an NH_3_ yield 1.72 times that of pristine g-C_3_N_5_ (Figure 17q). B-doping enhances light absorption and electron-hole separation and also provides B-O-H functional groups. The B site is capable of adsorbing and activating nitrogen, while the O-H site supplies the H^+^ to N_2_ for NH_3_ formation [37]. Moreover, Fe, an essential element of nitrogenase, plays an important role in N_2_ fixation; abundant oxygen vacancies (OVs) generated by W_18_O_49_ can adsorb and activate N_2_, and the narrow band gap of g-C_3_N_5_ expands the use of light. Based on these advantages, Li et al. [71] constructed Fe-W_18_O_49_/g-C_3_N_5_ hybrids for NH_3_ production at a rate of 131.6 mol·g^−1^·h^−1^, which was much superior to that of pristine g-C_3_N_5_ or Fe-W_18_O_49_.

### 4.4. NO Removal

**Figure 18:**
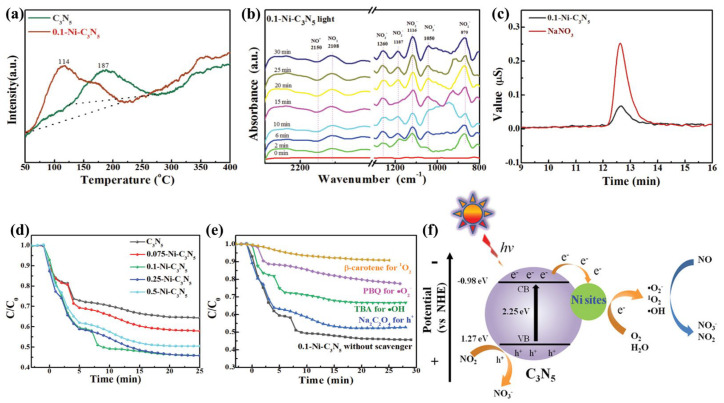
(**a**) O_2_-TPD spectra of C_3_N_5_ and 0.1−Ni−C_3_N_5_; (**b**) In situ DRIFTS spectra of 0.1−Ni−C_3_N_5_ on light irradiation; (**c**) Ion chromatography curves of NaNO_3_ and filtrate of reacted 0.1−Ni−C_3_N_5_ catalyst suspension; (**d**) Photocatalytic NO removal ratio for C_3_N_5_ and X−Ni−C_3_N_5_ catalysts; (**e**) Photocatalytic NO removal performance of 0.1−Ni−C_3_N_5_ in the presence of a series of trapping agents; (**f**) Proposed mechanism for photocatalytic NO removal on Ni-C_3_N_5_ material. Adapted with permission from [35]. Copyright Wiley-VCH GmbH, 2022.

Nitrogen oxides (NOx), especially NO, present a grave risk to global ecosystems. Photocatalysis is a viable NO removal technique that can convert NO to NO under visible light illumination. For example, Zhang et al. [60] constructed a Ni-C_3_N_5_ photocatalyst for NO removal. Under light irradiation, it increased O_2_ absorption (Figure 18a) and e^−^/h^+^ separation, promoting O_2_ activation and the production of more reactive oxygen species (ROS). In-situ DRIFT spectroscopy was used to investigate the conversion of NO to NO_3_^−^(Figure 18b). Ion chromatography was used to identify the formation of NO_3_^−^ (Figure 18c). Degradation experimental results show that 0.1-Ni-C_3_N_5_ has the largest NO removal efficiency (about 54%) (Figure 18d). A series of quenching experiments confirmed that the ROS (·O_2_^−^, ^1^O_2_, and ·OH) play an important role in NO removal (Figure 18e). The mechanism of NO removal by g-C_3_N_5_-based photocatalyst is further understood, as can be seen in Figure 18f. When photons with sufficient energy irradiate the Ni-C_3_N_5_, the electrons leap to the conduction band, forming holes in the valence band. The electrons can activate O_2_ to generate reactive oxygen species (·O_2_^−^, ^1^O_2_, and ·OH). The h^+^ and the generated ·O_2_^−^, ^1^O_2_, and ·OH can convert NO to NO_3_^−^. Moreover, TiO_2_ (P25)-C_3_N_5_ [72] Z-scheme heterojunctions displayed a NO removal rate of 67.1%, which is attributed to the effective separation of photogenerated e^−^ and h^+^ and the reserve of strong redox potentials provided by Z-scheme heterojunctions, promoting more ROS formation. Table 2 presents applications of g-C_3_N_5_-based materials for photocatalytic NO removal.

### 4.5. Pollutant Degradation

**Figure 19:**
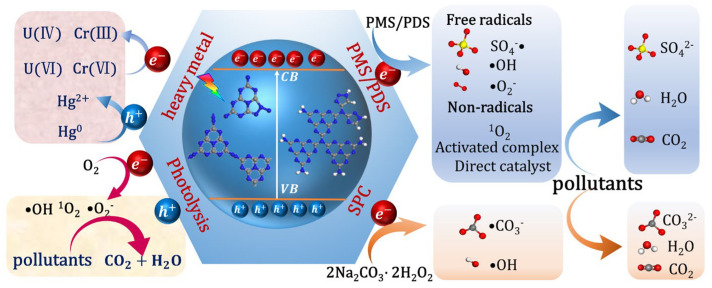
Photo-degradation processes of pollutants by g-C_3_N_5_-based materials.

Semiconductor materials based on g-C_3_N_5_ have been used extensively for the removal of different pollutants from water. Figure 19 summarizes the application of g-C_3_N_5_-based materials in the degradation of pollutants, including direct photocatalytic degradation and activated peroxide degradation. We first summarized the direct photocatalytic processes of the g-C_3_N_5_-based materials alone. When g-C_3_N_5_ is irradiated with photons of sufficient energy, electrons jump to the conduction band and generate holes in the valence band. Electrons oxidize O_2_ to generate ROS (·O_2_^−^, ^1^O_2_, ·OH), whereas holes oxidize H_2_O to generate ·OH. The resultant h^+^, ·O_2_^−^, ^1^O_2_, and ·OH may oxidize organic pollutants to form CO_2_ and H_2_O, while electrons can photo-reduce pollutants from high valence to low valence. For example, Cr (VI) and U (VI) can be reduced to Cr (IV) and U (IV), respectively [44,45,62,73]. Meanwhile, the h^+^ can also oxidize Hg^0^ to Hg^2+^ [43].

Moreover, persulfate (PMS/PDS) and sodium percarbonate (SPC) can be activated by g-C_3_N_5_-based materials to produce ROS for pollutant degradation. There are both free radicals and non-free radicals in the persulfate activation by g-C_3_N_5_-based materials. The free radical process refers to the oxidative degradation of pollutants by active species, such as SO_4_^−^·, ·OH, and ·O_2_^−^, produced by activating persulfate; the non-radical process includes the oxidation of pollutants by ^1^O_2_, electron transfer between persulfate and pollutants, and the direct oxidation of pollutants by persulfate. For example, Zhu et al. [28] demonstrated that g-C_3_N_5_ can activate PMS or PDS with the assistance of visible light to degrade emerging micropollutants. PMS can be activated by g-C_3_N_5_ to produce SO_4_^−^· and ·OH. Meanwhile, electrons in the g-C_3_N_5_ conduction band can react with O_2_ to produce ·O_2_^−^ and ^1^O_2_. The above species (h^+^, ·O_2_^−^, SO_4_^−^·, ^1^O_2_, and ·OH) are jointly involved in the degradation process of EMs. Ding et al. [39] demonstrated PDA-g-CN-1.0 obtained by coating g-C_3_N_5_ with polydopamine could effectively activate PMS without light to generate high-potential C-PMS* complexes, which could completely oxidize SMX within 5 min. Fang et al. [32] demonstrated that Co-C_3_N_5_ can activate PMS without light to completely degrade PCB28 within 30 min, which was significantly superior to the traditional metal-based activation. It was found that sulfate radical was the main active species for PCB28 degradation, which was generated from the decomposition of PMS activated by Co-N4. In contrast to this conclusion, Zhu et al. [33] discovered that high-valent cobalt oxides play an important role in SMX degradation by another Co-C_3_N_5_ activating PMS with the help of visible light. This difference may be caused by the combination of photocatalysis and the transition metal cobalt under visible light irradiation.

Sodium percarbonate (SPC, Na_2_CO_3_·1.5H_2_O_2_), as a solid carrier of H_2_O_2_, not only has the properties of H_2_O_2_, but also has the possibility of producing carbonate radicals. Due to its easy transportation, storage, and handling properties, it has been used as a substitute for H_2_O_2_ in AOPs. Zhang et al. [67] employed ultrathin-C_3_N_5_ and SPC coupling photocatalytic techniques to degrade sulfamethoxazole, arriving at a removal rate of 93.97% in 120 min. The main processes are as follows: (1) the separation of e^−^ and h^+^; (2) the formation of ROS (·OH, ·O_2_^−^, and ^1^O_2_); and (3) the degradation of pollutants by ROS (·OH, ·O_2_^−^, ^1^O_2_, ·CO_3_^−^, and h^+^). This work expands the application of g-C_3_N_5_-based materials in AOPs. Table 3 summarizes the recent developments in the photo-degradation and photo-reduction of pollutants by g-C_3_N_5_-based photocatalyst.

## 5. Conclusions and Future Outlook

In summary, this review presents an overview of the synthesis, functionalization, and applications of g-C_3_N_5_-based photocatalysts. Tremendous advancements have been witnessed during the past few years. Nevertheless, there are still some challenges and opportunities to be tackled.

Firstly, most reported g-C_3_N_5_-based photocatalysts are limited to the laboratory scale. To reach an industrial scale, time-saving and environmentally friendly processes for the synthesis of g-C_3_N_5_-based materials must be investigated. The new technology for the synthesis of g-C_3_N_5_ should be rapid, cost-effective, and time-efficient. Toxic templates, eco-damaging etchants, and complicated procedures should be avoided. Additionally, material recycling and sustainability are crucial elements for implementing industrial applications at scale. Combining g-C_3_N_5_ with narrower bandgap semiconductors can increase the efficiency of exploiting the full spectrum of sunlight; constructing suited heterojunctions can facilitate the separation of photoinduced carriers while preserving a high redox capacity; and coupling with magnetic materials can promote quick recycling.

Photocatalytic reactions heavily depend on separated electrons and holes. In order to comprehend the photocatalytic mechanism of the detection and characterization of electrons, holes, and reaction intermediates are vital. However, it is difficult to identify the transiently changing electrons, holes, and reaction intermediates using conventional characterization techniques. Therefore, more progressive technologies, such as in situ XPS, in situ IR spectra, and time-resolved terahertz spectra, may be employed to unveil the transitory processes of reactions.

As model pollutants for assessing photocatalytic activity, MB, MO, and RhB are often used. However, their photosensitivity and adsorption affect degradation efficiency, which is not an independent contribution of the photocatalysts. To accurately determine the photocatalytic efficacy of g-C_3_N_5_-based materials, it is necessary to employ other model pollutants, including medicines, pesticides, and phenols. In addition to elucidating the efficiency and processes of degradation, the biotoxicity and other possible dangers of degradation byproducts should be thoroughly explored.

With the further advancement of experimental research and theoretical calculations, the underlying photocatalysis process will be better understood, and the solar-to-energy conversion bottleneck might be overcome. Massive commercial applications of g-C_3_N_5_-based photocatalysts will foster renewable revolutions in the fields of energy and environmental engineering.

## Figures and Tables

**Table 1:** Summaries of g-C_3_N_5_ composite materials on photocatalytic H_2_ production.

Catalyst	Reaction Conditions	H_2_ Evolution (mmol·g^−1^·h^−1^)	Quantum Efficiency	Ref.
g-C_3_N_5_(MCN-8)	300 W Xe lamp (λ > 420 nm)	2.67		[18]
	Catalyst (1g L^−1^)			
	TEOA (10 vol%)			
	Pt cocatalyst			
Ultrathin C_3_N_5_ nanosheets	300W Xe lamp (λ > 420 nm)	0.03		[30]
	Catalyst (0.5 g L^−1^)			
	TEOA (10 vol%)			
NixSy-C_3_N_5_	300 W Xe lamp (λ > 420 nm)	35.44	37.0% (at 420 nm)	[63]
	Catalyst (1.5 g L^−1^)			
	TEOA (10 vol%)			
	Pt cocatalyst			
S-Ni (OH)_2_-C_3_N_5_	300 W Xe lamp (λ > 420 nm)	32.22	30.9% (at 420 nm)	[64]
	Catalyst (1.5 g L^−1^)			
	TEOA (10 vol%)			
	Pt cocatalyst			
Pt-C_3_N_5_	300 W Xe lamp (λ > 420 nm)	28.96	28.7% (at 420 nm)	[59]
	Catalyst (1.5 g L^−1^)			
	TEOA (10 vol%)			
CD/MoS_2_/C_3_N_5_	300 W Xe lamp (λ > 420 nm)	0.45		[65]
	Catalyst (0.5 g L^−1^)			
	Na_2_S and Na2SO3 (0.35 M)			
CdS/C_3_N_5_	300 W Xe lamp (λ > 420 nm)	0.50		[54]
	Catalyst (0.5 g L^−1^)			
	TEOA (10 vol%)			
	Pt cocatalyst (3 wt%)			
CdS/C_3_N_5_ (CCN)	300 W Xe lamp (λ > 420 nm)	2.69		[66]
	Catalyst (0.5 g L^−1^)			
	Na_2_S and Na2SO3 mixtures (0.35 M)			
	Pt cocatalyst (3 wt%)			
C_3_N_5_/Zn_0_._5_Cd_0_._5_S	300W Xe lamp(λ > 420 nm)	142.8	33.7% (at 420 nm)	[67]
	Catalyst (0.05 g L^−1^)			
	Na_2_S (0.35 M) and Na2SO3 (0.25 M)			
NH_2_-UiO-66/N-CN-2	visible light (λ ≥ 420 nm)	3.94	6.8% (at 420 nm)	[52]
	Catalyst (0.05 g L^−1^)			
	TEOA (10 vol%)			
	Pt cocatalyst (2 wt%)			
C_3_N_4_/rGO/C_3_N_5_	300 W Xe-lamp(λ ≥ 400 nm)	6.38	3.5% (at 420 nm)	[68]
	Catalyst (1 g L^−1^)			
	TEOA (10 vol%)			
	Pt cocatalyst (1.0 wt%)			

**Table 2:** Summary of applications in photocatalytic NO removal using g-C_3_N_5_-based materials.

Catalysts	Reaction Conditions	Removal Efficiency (%)	Reactive Species	Ref.
NixSy-C_3_N_5_	flow reactor and visible LED	40%	h^+^, ·OH, ·O_2_^−^, and ^1^O_2_	[63]
S-Ni (OH)_2_-C_3_N_5_	25 mg catalyst NO (~600ppb at 1000 mL min^−1^) A 30 W visible LED	42%	h^+^, ·OH, ·O_2_^−^, and ^1^O_2_	[64]
TiO_2_(P25)-C_3_N_5_	300 W Xe lamp (λ > 400 nm) 20 mg catalyst; NO: 450 ppb and RH: 15%	67 %	e^−^, h+, and ·O_2_^−^	[72]
Ni-C_3_N_5_	NO:600 ppb	54%	·OH, ·O_2_^−^, and ^1^O_2_	[60]

**Table 3:** Summary of the application of g-C_3_N_5_-based materials in degrading pollution.

Catalyst	Pollutants	Reaction Conditions	Reactive Species	Removal Efficiency	Ref.
RN-g-C_3_N_5_	MB	50 W halogen tungsten lamp	·O_2_^−^, ·OH	98%, 120 min	[20]
		Catalyst (1 g L^−1^)			
		MB (20 mL, 20.0 mg L^−1^)			
Ultrathin C_3_N_5_ nanosheets	MB	300 W Xe lamp (400 nm)	·O_2_^−^, h^+^	95%, 40 min	[30]
		Catalyst (0.5 g L^−1^)			
		MB solution (40 mL, 2.5 mg L^−1^)			
Nv g-C_3_N_5_-0.1	MB	50 W halogen tungsten lamp	·O_2_^−^, ·OH	95%, 120 min	[25]
	RhB	Catalyst (1 g L^−1^)		97%, 120 min	
	MO	Pollutant (20 mL, 40.0 mg L^−1^)		95%, 120 min	
CDs/MoS_2_/C_3_N_5_	MB	300 W Xe lamp (λ > 420 nm)	^1^O_2_, ·O_2_^−^, ·OH	94%, 120 min	[65]
		Catalyst (0.02 g L^−1^)			
		MB (50 mL, 30 mg L^−1^)			
CdS-MHP	RhB	Solar simulator (100 mW/cm^2^)	·O_2_^−^, ·OH, HO_2_, and e^−^	77%, 20 min 90%, 80 min	[53]
		Catalyst (0.1 g L^−1^)			
		RhB (50 mL, 0.01mM)			
g-C_3_N_5_/g-C_3_N_4_	RhB TC-HCl	300 W Xe lamp (λ > 420 nm)	^1^O_2_, ·O_2_^−^, and ·OH	98%, 30 min 92%, 60 min	[55]
		Catalyst (0.4 g L^−1^)			
		RhB (10 mg L^−1^, 50 mL)			
		TC-HCl (10 mg/L, 50 mL)			
g-C_3_N_5_/MIL-101(Fe)/PANCMA	Carbamazepine	300 W Xe lamp (λ >420 nm)	h^+^, ·O_2_^−^, and ·OH	94%, 40 min	[74]
	ciprofloxacin	Catalyst (0.1 g L^−1^)		97%, 40 min	
	tetracycline	Carbamazepine (50 mL, 200 ng mL^−1^)		98%, 40 min	
AgCl/g-C_3_N_5_	RhB	A halogen lamp (300W)	O_2_^−^, h^+^	96%, 30 min	[49]
		Catalyst (1 g L^−1^)			
		RhB solution (50 mL, 10 mg L^−1^)			
Er^3+^/Tb^3+^@BiOBr-g-C_3_N_5_	sulfamethoxazole	500 W tungsten halogen lamp	·O_2_^−^, ·OH	94%, 60 min	[75]
		Catalyst (1.3 g L^−1^)			
		SMX (75 mL, 10 ppm)			
g-C_3_N_5_/Bi_4_O_5_Br_2_	sulfathiazole (STZ)	300 W Xe lamp	·O_2_^−^, h^+^, and ·OH	100%, 60 min	[42]
		Catalyst (0.5 g L^−1^)			
		STZ (200 mL, 10 mg L^−1^)			
Ag_3_PO_4_/C_3_N_5_	TCH	A 300 W Xe lamp (λ > 400 nm)	·O_2_^−^ and ·OH	91%, 60 min	[47]
		Photocatalyst (1 g L^−1^)			
		TCH (50 mL, 20 mg L^−1^)			
C_3_N_5_/Ag_2_CO_3_	MB TC-HCl	300 W xenon lamp, λ > 400 nm	·O_2_^−^ and h^+^	97%, 90 min 98%, 100 min	[48]
		Catalyst (1 g L^−1^)			
		MB (60 mg L^−1^, 50 mL, and pH = 8.0)			
		TC-HCl (50 mg L^−1^, 50 mL, and pH = 4.8)			
Bi_2_WO_6_/g-C_3_N_5_	Tetracycline 2-ercaptobenzothiazol chlorpyrifos	under visible light (λ > 400 nm)	h^+^, ·O_2_^−^, and ·OH	93%, 90 min	[16]
		Catalyst (0.6 g L^−1^)		97%, 90 min	
		Pollutant (10 mg L^−1^, 50 mL)		94%, 90 min	
FeOCl/g-C_3_N_5_	TC	500 W Xe lamp (λ > 420 nm)	500 W Xe lamp (λ > 420 nm)	95%, 40 min	[50]
		Catalyst (1 mg mL^−1^)			
		TC (75 mL, 10 mg L^−1^)			
		H2O2 solution (30%, 200 μL)			
CeTi_2_O_6_/g-C_3_N_5_	2,4 dichlorophenol	300 W xenon lamp (λ > 420 nm)	·O_2_^−^, ·OH	96%, 120 min	[46]
		Photocatalyst (1.6 g L^−1^)			
		2,4-DCP solution (75 mL, 10 ppm)			
C_3_N_5_@NH_2_-MIL-125	RhB	300 W xenon lamp (λ > 420 nm)	·O_2_^−^, h^+^, and ·OH	93%, 120 min	[76]
		Catalyst (0.5 g L^−1^)			
		RhB (100 mL, 10 mg L^−1^)			
2D/0D C_3_N_5_/ Bi_2_WO_6_	TC	300 W Xe lamp (λ > 420 nm)	·O_2_^−^, h^+^, and ·OH	94%, 60 min	[44]
		Catalyst (0.2 g L^−1^)			
		TC (20 mg L^−1^, 100 mL, and pH 5.2)			
2D/0D Bi_2_MoO_6_/C_3_N_5_	TC	300 W Xe lamp (λ > 420 nm)	·O_2_^−^, ·OH, and h^+^	88%, 75 min	[45]
		Catalyst (0.3 g L^−1^)			
		TC (20 mg L^−1^, 100 mL, and pH 5.2)			
2D/2D Bi2WO6@g-C_3_N_5_	TC	300 W Xe lamp (λ > 420 nm)	·O_2_^−^, ·OH, and h^+^	100%, 60 min	[77]
		Catalysts (0.4 g L^−1^)			
		TC (10 mg L^−1^, 50 mL)			
2D/2D Bi_4_O_5_Br_2_/g-C_3_N_5_	Ciprofloxacin bisphenol-A	Xe lamp at 500 W (165 mW/cm^2^)	·O_2_^−^, ·OH, and h^+^	94%, 60 min 92%, 80 min	[78]
		Catalysts (0.67 g L^−1^)			
		Pollutant (20 mg L^−1^, 75 mL)			
2D/0D C_3_N_5_/ Bi_2_WO_6_	Cr (VI)	300 W Xe lamp (λ > 420 nm)	·O_2_^−^ and e^−^	97%, 50 min	[44]
		Catalyst (0.2 g L^−1^)			
		Cr (VI) (10 mg L^−1^, 100 mL, and pH 2.5)			
2D/0D Bi_2_MoO_6_/C_3_N_5_	Cr (VI)	300 W Xe lamp (λ > 420 nm)	·O_2_^−^ and e^−^	97%, 60 min	[45]
		Catalyst (30 mg)			
		Cr (VI) (10 mg L^−1^, 100 mL, and pH 2.5)			
2D/2D C_3_N_5_/GO	U(VI)	300 W Xe lamp (λ > 420 nm)	e^−^	96%, 90 min	[56]
		Catalyst (0.5 g L^−1^)			
		U(VI) solution (10 ppm, 100 mL)			
2D/2D C_3_N_5_/RGO	U(VI)	300 W Xe lamp (λ > 420 nm)	e^−^	95%, 100 min	[62]
		Catalyst (0.2 g L^−1^)			
		U(VI) (10 mg L^−1^, 100 mL)			
		pH 5.0			
		T = 298 K			
Bi_4_O_5_I_2_/g-C_3_N_5_	Hg^0^	6 W LED lamp (λ > 400 nm)	·O_2_^−^ and h^+^	93%, 60 min	[43]
		Catalyst (40 mg)			
		Mercury vapors (65 μg m^−3^)			
		gas flow rate of 1.2 L min^−1^			
C_3_N_5_	SMX	300 W xenon lamp (λ > 420 nm)	·O_2_^−^, h^+^, SO_4_^−^⋅, ^1^O_2_, and ·OH	PMS/C3N5/Vis	[28]
		Catalyst (0.5 g L^−1^)		67%, 60 min	
		PMS (0.125 g L^−1^)		PDS/C3N5/Vis	
		SMX (5 mg L^−1^)		70%, 60 min	
PDA-g-CN-1.0	SMX	Catalyst (50 mg L^−1^)	C−PMS * complexes	100%, 20 min	[39]
		PMS (1mM)			
		SMX (10 mg L^−1^)			
Co-C_3_N_5_	PCB28	Catalyst (0.2 g L^−1^)	SO_4_^−^· and ·OH	96%, 30 min	[32]
		PMS (2.0 mM)			
		PCB28 (0.5 mg L^−1^)			
Co-C_3_N_5_	SMX	500 W Xe lamp (λ > 420 nm)	high-valent cobalt oxide (Co (IV)) species	100%, 20 min	[33]
		Catalyst (0.5 g L^−1^)			
		PMS (1.0 mM)			
		SMX solution (30 mL, 10 mg L^−1^)			
U-C_3_N_5_	SMZ	visible light (λ > 420 nm)	·OH, ·O_2_^−^, ^1^O_2_, ·CO_3_^−^, and h^+^	94%, 120 min	[79]
		Catalyst (0.4 g L^−1^)			
		SPC (0.1 g L^−1^)			
		SMZ (100 mL, 10 mg L^−1^)			

* indicates that C and PMS form a complex (C-PMS*)

## Data Availability

The data presented in this study are available in this article.
